# Somatic and intergenerational G4C2 hexanucleotide repeat instability in a human C9orf72 knock-in mouse model

**DOI:** 10.1093/nar/gkae250

**Published:** 2024-04-10

**Authors:** Nada Kojak, Junko Kuno, Kristina E Fittipaldi, Ambereen Khan, David Wenger, Michael Glasser, Roberto A Donnianni, Yajun Tang, Jade Zhang, Katie Huling, Roxanne Ally, Alejandro O Mujica, Terrence Turner, Gina Magardino, Pei Yi Huang, Sze Yen Kerk, Gustavo Droguett, Marine Prissette, Jose Rojas, Teodoro Gomez, Anthony Gagliardi, Charleen Hunt, Jeremy S Rabinowitz, Guochun Gong, William Poueymirou, Eric Chiao, Brian Zambrowicz, Chia-Jen Siao, Daisuke Kajimura

**Affiliations:** Regeneron Pharmaceuticals, Tarrytown, NY 10591, USA; Regeneron Pharmaceuticals, Tarrytown, NY 10591, USA; Regeneron Pharmaceuticals, Tarrytown, NY 10591, USA; Regeneron Pharmaceuticals, Tarrytown, NY 10591, USA; Regeneron Pharmaceuticals, Tarrytown, NY 10591, USA; Regeneron Pharmaceuticals, Tarrytown, NY 10591, USA; Regeneron Pharmaceuticals, Tarrytown, NY 10591, USA; Regeneron Pharmaceuticals, Tarrytown, NY 10591, USA; Regeneron Pharmaceuticals, Tarrytown, NY 10591, USA; Regeneron Pharmaceuticals, Tarrytown, NY 10591, USA; Regeneron Pharmaceuticals, Tarrytown, NY 10591, USA; Regeneron Pharmaceuticals, Tarrytown, NY 10591, USA; Regeneron Pharmaceuticals, Tarrytown, NY 10591, USA; Regeneron Pharmaceuticals, Tarrytown, NY 10591, USA; Regeneron Pharmaceuticals, Tarrytown, NY 10591, USA; Regeneron Pharmaceuticals, Tarrytown, NY 10591, USA; Regeneron Pharmaceuticals, Tarrytown, NY 10591, USA; Regeneron Pharmaceuticals, Tarrytown, NY 10591, USA; Regeneron Pharmaceuticals, Tarrytown, NY 10591, USA; Regeneron Pharmaceuticals, Tarrytown, NY 10591, USA; Regeneron Pharmaceuticals, Tarrytown, NY 10591, USA; Regeneron Pharmaceuticals, Tarrytown, NY 10591, USA; Regeneron Pharmaceuticals, Tarrytown, NY 10591, USA; Regeneron Pharmaceuticals, Tarrytown, NY 10591, USA; Regeneron Pharmaceuticals, Tarrytown, NY 10591, USA; Regeneron Pharmaceuticals, Tarrytown, NY 10591, USA; Regeneron Pharmaceuticals, Tarrytown, NY 10591, USA; Regeneron Pharmaceuticals, Tarrytown, NY 10591, USA; Regeneron Pharmaceuticals, Tarrytown, NY 10591, USA

## Abstract

Expansion of a G_4_C_2_ repeat in the *C9orf72* gene is associated with familial Amyotrophic Lateral Sclerosis (ALS) and Frontotemporal Dementia (FTD). To investigate the underlying mechanisms of repeat instability, which occurs both somatically and intergenerationally, we created a novel mouse model of familial ALS/FTD that harbors 96 copies of G_4_C_2_ repeats at a humanized *C9orf72* locus. In mouse embryonic stem cells, we observed two modes of repeat expansion. First, we noted minor increases in repeat length per expansion event, which was dependent on a mismatch repair pathway protein Msh2. Second, we found major increases in repeat length per event when a DNA double- or single-strand break (DSB/SSB) was artificially introduced proximal to the repeats, and which was dependent on the homology-directed repair (HDR) pathway. In mice, the first mode primarily drove somatic repeat expansion. Major changes in repeat length, including expansion, were observed when SSB was introduced in one-cell embryos, or intergenerationally without DSB/SSB introduction if G_4_C_2_ repeats exceeded 400 copies, although spontaneous HDR-mediated expansion has yet to be identified. These findings provide a novel strategy to model repeat expansion in a non-human genome and offer insights into the mechanism behind *C9orf72* G_4_C_2_ repeat instability.

## Graphical Abstract

**Graphical Abstract:**
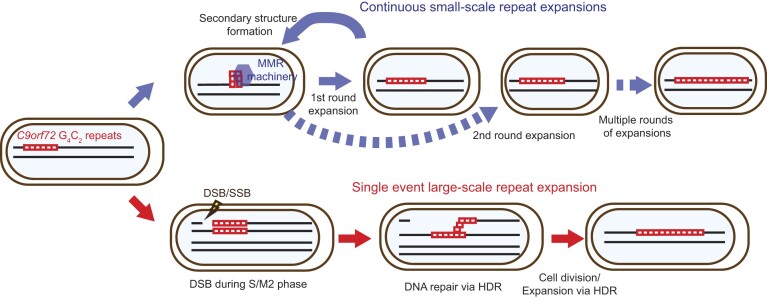

## Introduction

More than 3% of the human genome consists of short tandem repeats (STRs), also referred as simple sequence repeats (SSRs) or microsatellites (1). Expansion of STRs at specific genomic locations is associated with approximately 50 human diseases known as repeat expansion disorders (REDs) (2,3). Despite the diversity of STR sequences and resulting disease pathologies, the STR length is a key factor for disease severity and age-of-onset in many REDs. Therefore, studying how disease-causing STRs expand or contract in the genome, i.e. repeat instability, is indispensable for the understanding of REDs.

Broadly speaking, there are two types of repeat instability that change the length of disease-causing STRs: Intergenerational repeat instability and somatic repeat instability (4–6). Intergenerational repeat instability refers to the changes that occur during parent-child transmission. In this process, major changes in repeat length, sometimes more than ten-fold increases, have been observed. Intergenerational repeat expansions have been well documented in some REDs. Normal individuals typically possess 5–40 copies of CGG repeats in the 5′-UTR of the *FMR1* gene. Expanded CGG repeats with 55–200 copies, which are associated with fragile X-associated tremor/ataxia syndrome (FXTAS) and fragile X–associated primary ovarian insufficiency (FXPOI), are unstable and frequently expand intergenerationally, through maternal inheritance, resulting in more than 200 copies of CGG repeats that exhibit fragile X syndrome (FXS) (7–11). Another exemplar RED, myotonic dystrophy type 1 (DM1) is caused by CTG repeat expansion located in the 3′ untranslated region of the *DMPK* gene (12,13). While CTG repeat expansions can occur through both maternal and paternal inheritance, the symptom-associated mutation with more than 80 copies of CTG repeats exhibit a strong bias toward larger expansions when inherited from the mother (13–16).

Despite well-known human genetics studies, underlying molecular mechanisms involved in the expansion of disease-causing STRs across generations are still not well characterized. In general, RED mouse models with STRs comparable to human pathogenic repeat length do not recapitulate human intergenerational repeat instability (2,4). For instance, DM1-associated CTG repeats in humans can expand more than 10-fold between generations, but changes of this magnitude are rarely observed in DM1 mouse models (17–19).

The second type of repeat instability is somatic repeat instability (5,20), which is well explained by continuous small-scale changes caused by DNA mis-repair or mis-replication that accumulate in the genome over time. Since DNA metabolism greatly varies among cell types, somatic repeat instability exhibits a tissue-specific pattern (5). In contrast to our poor understanding of intergenerational repeat instability, studies have revealed key molecular players involved in somatic repeat instability. Proteins in the DNA mismatch repair (MMR) pathway have been best characterized for its role in this regard (21,22). In RED mouse models for Huntington's disease (HD), DM1, Friedreich's ataxia (FA), or FXS, blockade of the MMR pathway by inactivating genes such as *Msh2*, *Msh3* or *Mlh1*, dramatically reduced somatic repeat instability (23–34). Consistent with the findings from mouse models, human genome-wide association studies (GWAS) identified the genes in this pathway, including *MLH1*, *MSH3* or *PSM2*, as genetic modifiers for disease onset in HD (35,36). Notably, *MSH3* is a common modifier for HD as well as DM1 (37), further supporting the notion that proteins in the MMR pathway play a key role in somatic instability and disease pathogenesis.

Although the major change in STR length during parent-child transmission in RED patients can be explained as a sum of continuous small-scale expansions, it is possible that other mechanisms may exist to achieve the large-scale intergenerational repeat expansions (2). Alternative mechanisms, including homology-directed repair (HDR)-dependent pathway, were proposed to explain this magnitude of changes as a single event, or with a limited number of events, although these hypotheses have not been explored in depth (6,38). Kim *et al.* showed that Break-Induced Replication (BIR), one of the HDR pathways to repair one-ended DSBs, was involved in large-scale CAG repeat expansions that were independent from the continuous small-scale repeat expansions in the yeast system (39), while other studies showed association of *FMR1* CGG repeat instability and BIR in mammalian genomes (40,41). To date, the contribution of homologous recombination toward repeat instability is just beginning to be examined.

Expansion of the G_4_C_2_ hexanucleotide repeat in the *C9orf72* gene causes Amyotrophic Lateral Sclerosis-Frontotemporal Dementia (ALS-FTD) disease spectrum (42–44). Alleles with >30 copies of G_4_C_2_ repeats are considered risk alleles, and alleles containing hundreds to thousands of copies are frequently found in autopsy samples. Like other disease-causing STRs, it is difficult to obtain precise sequence over the G_4_C_2_ repeats. In addition, 100% GC content in DNA sequence, as well as the large expansions often found in patients make it very difficult to determine the precise *C9orf72* G_4_C_2_ repeat length. Furthermore, there is high heterogeneity in the G_4_C_2_ repeat instability among patients (45–47). Accordingly, intergenerational and somatic repeat instability of the *C9orf72* G_4_C_2_ repeats has not been systematically analyzed, and hence phenotype-genotype relationships have been poorly defined (47,48). Efforts have been made to generate mouse models for *C9orf72*-associated ALS/FTD using patient-derived Bacterial Artificial Chromosomes (BAC) (49–52), but thus far such mouse models have not recapitulated the full spectrum of disease pathology observed in humans. The lack of animal models that faithfully recapitulate *C9orf72* repeat expansion-dependent phenotypes is a significant gap for the understanding of the disease and hence the development of therapeutics.

Compounding the difficulty in reproducing human STR dynamics, engineering an animal model with even an unexpanded STR is technically challenging. Inserting mutant sequences into the genome of embryonic stem cells by homologous recombination using plasmid-based targeting vectors has been the most common procedure to generate animal models with large mutations (53). STRs beyond a certain length are typically unstable in bacteria in which mutant DNA fragments are routinely propagated in the laboratory (49,54–56). The instability of STRs in bacteria makes it difficult to generate animal models for REDs, especially those with very large STRs.

In this study, we generated humanized *C9orf72* alleles with pathogenic length of G_4_C_2_ repeats and studied the molecular characteristics of repeat instability. We found (i) 96 copies of *C9orf72* G_4_C_2_ repeats exhibited minor repeat instability in cultured mouse embryonic stem (mES) cells and somatic tissues in unperturbed conditions; (ii) a double-strand break (DSB) or a single-strand break (SSB) proximal to the repeats induced large-scale expansions of the *C9orf72* G_4_C_2_ repeats and two other STRs associated with RED; (iii) these large-scale repeat expansions occurred in a HDR-dependent manner; (iv) the G_4_C_2_ repeats exhibited a repeat length-dependent intergenerational instability in mice. These findings provided us key insights into the molecular evolution of the *C9orf72* G_4_C_2_ repeat expansion in the genome.

## Materials and methods

### Reagents

Synthetic DNA fragments were generated and cloned into plasmids by Azenta Life Sciences (South Plainfield, USA), Genscript Biotech Crop (Piscataway, USA), or Thermo Fisher Scientific (Waltham, USA). All the oligonucleotides and gRNAs were synthesized by Integrated DNA Technologies, Inc (Coralville, USA). All the gRNA sequences (protospacer + PAM) and oligonucleotide sequences are listed in Supplementary Table S1 and Supplementary Table S2. All the gRNAs were synthesized as sgRNA in this study. WT Cas9 and Cas9-D10A nickase were purchased from Integrated DNA Technologies, Inc. Taqman assays were purchased from LGC, Biosearch Technology (Hoddesdon, UK). siRNAs against *Rad51* (L-0627300-00), *Pold3* (L-046305-01), and control non-target siRNA (D-001810-01) were purchased from Horizon Discovery Biosciences (Teddington, UK). Anti-Msh2 and Anti-Rad51 antibodies were purchased from Cell Signalling Technology, Inc (Danvers, USA). Anti-Pold3 antibody (21935-1-AP) was purchased from Proteintech (Rosemont, USA). Anti-b-actin antibody was purchased from Sigma Aldrich (St. Louis, USA). Anti-mouse secondary antibody and anti-rabbit secondary antibody were purchased from Protein Simple (San Jose, USA).

### Biological resources

F1H4 ES cell line derived from 129/Sv:C57BL/6 (50%:50%) background was used in this study (57). C57BL/6NTac and Swiss Webster mice were purchased from Taconic Biosciences (Rensselaer, USA). PRCI-11 human BAC library and PRCI-23 mouse BAC library derived from C57BL/6J (58,59) were purchased from Thermo Fisher Scientific. 129Sv-derived BAC library was purchased from Sanger Institute (60). B-lymphocytes derived from a FA patient that harbour 1030 and 650 copies of GAA repeats (GM15850) was obtained from Coriell Institute (Camden, USA).

### Mutant alleles

**Figure 1.**
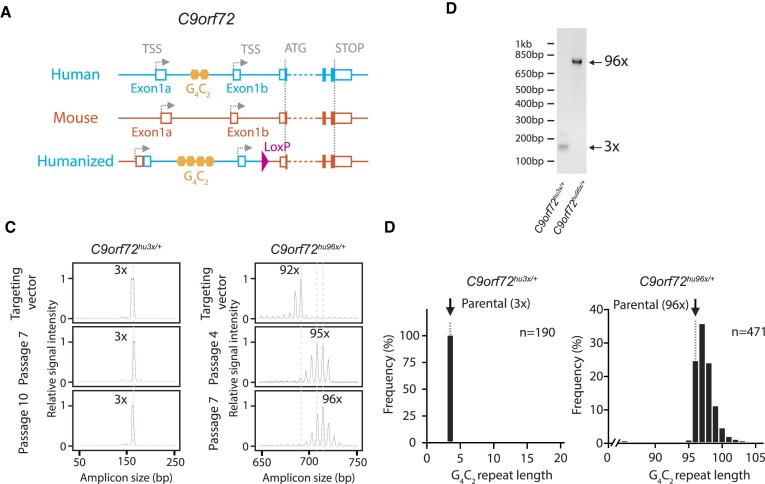
Humanized *C9orf72* alleles. (**A**) Humanized *C9orf72* allele schematic. Orange: mouse; blue: human; yellow hexagons: G_4_C_2_ repeats. TSS: Transcription start site. (**B**) 2-primer gene-specific PCR analysis of the G_4_C_2_ repeats in targeted ES cells. (**C**) RP-PCR analysis of the targeting vectors, *C9orf72*^hu3x/+^ ES cells (passages 7 and 10), *C9orf72*^hu96x/+^ ES cells (passages 4 and 7). Capillary electrophoresis (CE) traces of gene-specific PCR products shown. (**D**) The G_4_C_2_ repeat length after a single subcloning *C9orf72*^hu3x/+^ ES cells and *C9orf72*^hu96x/+^ ES cells. *n* = 190 and *n* = 471 subclones analyzed respectively. Parental repeat lengths shown as dotted lines.

The control 3× G_4_C_2_ (3 copies of G_4_C_2_) humanized *C9orf72* allele was generated as follows. A DNA fragment that contained a part of mouse exon1a (chr4: 35226073–35226172 mm10) human exon1a, human intron downstream of exon1a, exon1b, intron downstream of exon 1b (chr9:27573740–27572796, hg38), and a part of mouse intron downstream of exon 1b (chr4: 35225210–35225219 mm10) was synthesized and cloned into a plasmid that contained the replication origin of Colicin E1 (ColE1) and Ampicillin drug resistant gene (Supplementary Figure S1A-i). AsiSI restriction enzyme sites were inserted both upstream and downstream of the 3× G_4_C_2_ for the cloning processes. Similarly, XhoI and NheI sites were inserted between 3′ human-mouse junction. A floxed Neomycin drug resistant cassette was inserted into the 3′ human–mouse junction by ligation (Supplementary Figure S1A-ii). The human sequence and the drug resistant cassette in this construct were transferred into mouse BAC (RP23-434N2) using bacterial homologous recombination (BHR) (61) (Supplementary Figure S1A-iii). This BAC-based targeting vector (Supplementary Figure S1A-iv) was used for gene targeting in F1H4 ES cells to obtain the humanized *C9orf72* with 3× G_4_C_2_ following VelociGene® method (57). The floxed Neomycin cassette was removed from the genome by electroporating Cre recombinase mRNA (TriLink, San Diego, USA) into the targeted ES cells. These targeted-, cassette removed ES cells are referred as *C9orf72^hu3x/+^* ES cells. To generate repeat-expanded G_4_C_2_ alleles, a DNA fragment that contained 50× G_4_C_2_, and type IIS restriction enzyme sites BsmbI and BsaI at 5′ and 3′ adjacent to the repeats respectively was synthesized (Thermo Fisher Scientific), and then cloned into a plasmid with ColE1 replication of origin and ampicillin (Supplementary Figure S1B-i). The propagation of all the plasmids that contained expanded G_4_C_2_ repeats was carried out in Stbl4^TM^ E. Coli strain (Thermo Fisher Scientific) at 30°C or at room temperature. The plasmid was digested either by BsmbI and XmnI (New England Biolabs, Ipswich, MA) that cleaved the plasmid backbone once, or BsaI and XmnI (Supplementary Figure S1B-ii). Two repeat-containing fragments were ligated together to obtain a novel plasmid that contained 100x G_4_C_2_ (Supplementary Figure S1B-iii). A Spectinomycin drug resistant cassette was then inserted at 3′ downstream of the 100× G_4_C_2_ by ligation (Supplementary Figure S1B-iv). Since we failed to generate a BAC-based targeting vector that contained 100× G_4_C_2_ due to the bacterial instability, instead, we generated a small plasmid-based targeting vector using a plasmid backbone that contained ColE1 replication of origin. To generate the targeting vector, following five DNA fragments were ligated together (Supplementary Figure S1B-v). (a) 5kb 5′ mouse homology arm and human sequence 5′ upstream of the G_4_C_2_ repeats (chr4: 35226073–35231444) that was PCR amplified from the BAC-based 3× G_4_C_2_ targeting vector described above. (b) 100× G_4_C_2_ with Spectinomycin resistant cassette. (c) human sequence corresponding to 3′ downstream of the G_4_C_2_ repeats and 5′ part of floxed neomycin cassette. (d) 3′ part of floxed neomycin cassette and 5 kb 3′ mouse homology arm (ch4: 35220046–35225219) amplified from the 3× G_4_C_2_ targeting vector. (e) A plasmid backbone with ColE1 replication origin and Ampicillin. All three validation methods, 3-primer RP-PCR, Sanger sequencing, and STRique analysis using nanopore sequencing reads (explained below at Nanopore sequencing/STRique analysis sub-section below) confirmed that the most abundant specie in the final targeting vector contained 92 copies of G_4_C_2_ repeats (Figure 1C, Supplementary Figure S1D, and Supplementary Figure S2). Gene targeting was performed in the same manner with the BAC-based 3× G_4_C_2_ targeting vector. We obtained two distinct targeted clones that had either 31× G_4_C_2_ or 96× G_4_C_2_ repeats. Drug resistant cassettes were removed in the same manner with *C9orf72^hu3x/+^* mES cell line. As described in the results section, 31x G_4_C_2_ repeats were stable over several passages in culture (Supplementary Figure S4C). These cells were referred as *C9orf72^hu31x/+^* in the main text. G_4_C_2_ repeat copy number kept changing in the 96× G_4_C_2_ mES cells (Figure 1C and D). Therefore, for most of the experiments in this study, we used mES cell clones that contained 95–97 copies of G_4_C_2_ repeats as 96× G_4_C_2_ mES cells (Figure 1C). These ES cells were referred as *C9orf72^hu96x/+^* in the main text. In analysis, we called the copy number of the most abundant G_4_C_2_ repeat species in a given sample as ‘repeat length’.

To generate larger G_4_C_2_ repeat alleles in mES cells, either WT Cas9 and C9-5′ gRNA alone, or WT Cas9 and both C9-5′ and C9-3′ gRNAs (dual gRNA expansion), were electroporated as ribonucleoproteins (RNPs) into *C9orf72^hu96x/+^* mES cells (Figure 8A and B). After the electroporation, clones were isolated, grown in 96-well cell culture plates, and the G_4_C_2_ repeat length was screened by 2-primer gene-specific PCR. The largest expansion clone that had approximately 250 copies of G_4_C_2_ repeats generated by single C9-5′ gRNA introduction was chosen for detailed analysis (*C9orf72^hu250x/+^* ES cells). Also, the largest expansion clone generated from dual gRNA expansion that had approximately 300 copies G_4_C_2_ was subjected to further analysis (*C9orf72^hu300x/+^* ES cells). The 250× G_4_C_2_ repeats in *C9orf72^hu250x/+^* mES cell clone was further expanded by electroporating WT Cas9 and C9-3′ gRNA as RNPs. Three repeat-expanded clones were chosen: 400 copies of G_4_C_2_ repeats clone (*C9orf72^hu400x/+^* ES cells), 450 copies of G_4_C_2_ repeats clone (*C9orf72^hu450x/+^* ES cells) and 550 copies of G_4_C_2_ repeats clone (*C9orf72^hu550x/+^* ES cells). The repeat expansion was confirmed by Southern blotting and STRique analysis using nanopore sequencing reads (Figure 8C and D). All the Cas9 cleavage sites in these repeat-expanded clones were intact except the 3′-DSB site in *C9orf72^hu300x/+^* ES cell clone as shown in Supplementary Figure S9A and Supplementary Figure S9B.

*Msh2* ^−/−^ and *Pif1*^−/−^ alleles were generated by electroporating four gRNAs per gene (two 5′ upstream and two 3′ downstream gRNAs (Supplementary Figure S5C, Supplementary Figure S7H, and Supplementary Table S1) together with WT Cas9 into *C9orf72*^hu96x/+^ mES cell clone to excise out the entire gene body; *Msh2* (chr17: 87672608–87712856, mm10) and *Pif1* (Chr9: 65586984–65596166), respectively. The correctly targeted clones were selected by Taqman quantitative PCR assay (57). At both loci, copy number assays confirmed the removal of the entire gene using three internal assays (probes 2, 3 and 4) without extensive deletion outside of the gene body using two external assays (probe 1 and 5) as shown in Supplementary Figure S5C, D and Supplementary Figure S7H, I. Sequences of Taqman assay primers and probes are listed in Supplementary Table S3.

Humanized *Tcf4* allele with 60 copies of CTG repeats were generated as follows. A synthetic DNA fragment that contained human *TCF4* intron 2, its surrounding sequences (Chr 18:55585008–55587009, hg38), 60 copies of CTG repeats, mouse homology arm sequences (Chr 18:69347306–69347405, and Chr18:69349426–69349525, mm10) for BHR, was synthesized and cloned into a plasmid with ColE1 replication of origin and Ampicillin drug resistant gene (Supplementary Figure S5A-i). For the cloning purpose, restriction enzyme sites XhoI and NheI were inserted at the human/mouse 3′ junction. After inserting Neomycin drug resistant cassette by ligation (Supplementary Figure S5A-ii), the human sequence and the drug resistant cassette was transferred into a mouse BAC (RP23-163D18) by BHR (Supplementary Figure S5A-iii). The modified BAC (Supplementary Figure S5A-iv) was used as a targeting vector to generate the repeat-expanded humanized *Tcf4* allele following VelociGene® method. The 60 copies of CTG repeats in mES cells were stable during the passages we used in this study.

Humanized *Fxn* allele with 400 copies of GAA repeats was generated as follows. A DNA fragment that contained spectinomycin drug resistant gene, a SgrDI restriction enzyme site, and a part of human *FXN* intron 1 sequence (Chr9: 69036147–69036346, hg38) was synthesized. Another DNA fragment that contained human sequence downstream of *FXN* gene (Chr 9:69079178–69079377, hg38), floxed Neomycin drug resistant cassette and an I-CeuI enzyme site, was synthesized. These two DNA fragments were inserted into a human BAC (RP11-912J3) at 5′ upstream and 3′ downstream of *FXN* gene locus respectively by BHR (Supplementary Figure S5B-i). A DNA fragment that contained mouse *Fxn* 5′ sequence (Chr19: 24280566–24280765, mm10), human *FXN* exon1 coding sequence and a part of human *FXN* intron 1 (Chr9: 69035783–69036146, hg38), a SgrDI enzyme site, Hygromycin drug resistant gene, and an I-CeuI enzyme site, was synthesized and inserted into a mouse BAC (bMQ-301N7) to remove *Fxn* coding sequence (Chr19: 24261903–24280565, mm10) (Supplementary Figure S5B-iii). A SgrDI/I-CeuI fragment in the modified human BAC that contained *FXN* gene except exon1 and a part of intron 1 (Chr9: 69036147–69076646, hg38) and floxed Neomycin cassette was ligated into the modified mouse BAC SgrDI/I-CeuI sites (Supplementary Figure S5B-iv). This modified BAC (Supplementary Figure S5B-v) was used as a targeting vector to generate humanized *Fxn* allele in mES cells following VelociGene® method. To generate repeat-expanded humanized Fxn alleles, a plasmid-based targeting vector was generated by ligating following three DNA fragments: (a) a 650 copies of GAA repeats-containing PCR product amplified from the patient-derived cells (GM15850, Coriell Institute) using primers Fxn-1F and Fxn-1R, (b) a Puromycin resistant cassette harboured by two Rox sequences, and (c) a plasmid that contained 5′ homology arm (Chr9: 69035783–69036989, hg38), 3′ homology arm (Chr9: 69036989, hg38), a plasmid backbone that contained ColE1 replication of origin and Ampicillin drug resistant gene (Supplementary Figure S5B-vii). The resulting plasmid-based targeting vector (Supplementary Figure S5B-viii) was electroporated into the humanized *Fxn* ES cell clone above to generate repeat-expanded humanized *Fxn* allele. None of the targeted clone retained the full 650 copies of GAA repeats. We chose one of the targeted clones that retained 400 copies of GAA, and then removed the drug resistant cassettes from this allele via Dre recombinase. This targeted and cassette-removed clone (Supplementary Figure S5B-x) was used in this study. The 400 copies of GAA repeats in mES cells were stable during the passages we used in this study.

A *Rosa26*-Cas9 targeting vector was generated in the same manner as previously described (62), except that WT Cas9 was inserted instead of dCas9-SAM.

### Mice

For the intergenerational repeat instability study, F0 VelociMice were created from *C9orf72*^hu400x/+^ and *C9orf72*^hu550x/+^ ES cell clones. These F0 mice were crossed with C57BL/6NTac WT mouse to obtain F1 generation. Generations were referred as filial generation. For each line (400× G_4_C_2_ line and 550x G_4_C_2_ line), resulting F1 mice were inter-crossed to establish separate colonies. Additional lines were obtained from spontaneous repeat contractions during the colony maintenance. A repeat-contracted male mouse that had approximately 250 copies of G_4_C_2_ repeats (heterozygous, *C9orf72*^hu250x/+^) and a repeat-contracted female mouse that had approximately 300 copies of G_4_C_2_ repeat (heterozygous, *C9orf72*^hu300x/+^) were isolated from the 400× G_4_C_2_ line. Two repeat-contracted female mice that had approximately 250 copies of G_4_C_2_ (homozygous, *C9orf72*^hu250x/hu250x^) were isolated from the 550× line. For testing intergenerational repeat instability, following breeding pairs were set up and the repeat length in the resulting offspring tails was analyzed. 96× G_4_C_2_ line: F0 *C9orf72*^hu96x/+^ mice (three male and three female) generated by IVF were intercrossed with *C9orf72*^+/+^ mice. 250×–300× G_4_C_2_ line: One F4 *C9orf72*^hu250x/+^ male mouse, two F4 *C9orf72*^hu250x/hu250x^ female mice, and one F4 *C9orf72*^hu300x/+^ female mouse described above as ‘repeat-contracted’ mice were intercrossed with *C9orf72*^+/+^ mice. 400× G_4_C_2_ line: Four F4 *C9orf72*^hu400x/+^ male and three F4 female *C9orf72*^hu400x/+^ mice were intercrossed with *C9orf72*^+/+^ mice. 550× G_4_C_2_ line: One male and two female F4 *C9orf72*^hu550x/+^ mice, and one F4 female *C9orf72*^hu550x/hu550+^ mice were intercrossed with *C9orf72^+/+^* mice.

100% ES derived F0 *C9orf72*^hu96x/+^*; Msh2*^−/−^ mice were generated from the corresponding mES cells. *C9orf72*^hu96x/+^*; Msh2*^−/−^ tissues were collected at 2 months of age. All animals were housed under a 14-h light/10-h dark cycle (light from 05:00 to 19:00) with *ad libitum* access to food and water. All the procedures were conducted in compliance with protocols approved by the Institutional Animal Care and Use Committee of Regeneron Pharmaceuticals.

VelociMouse® method (63) was used to generate 100% ES-derived F0 mice. For Cas9-D10A nickase one-cell embryo injection experiments, C57BL/6NTac female mice were super-ovulated by the intraperitoneal administration of 5 IU of PMGS (ProSpec Protein Specialists, Rehovot, Israel), followed by the intraperitoneal administration of 5 IU hCG (ProSpec Protein Specialists) 48 hour later. The cumulus–oocyte complexes were collected in Cook's MEDIUM^®^ (COOK Medical LLC, Bloomington, USA), preincubated in FERTIUP^®^ Mouse Sperm Preincubation Medium (Cosmo Bio USA, Carlsbad, USA), inseminated with *C9orf72^hu96x/+^* sperm for about 1 h, and then incubated at 37°C in 5% CO_2_ and 5% O_2_ with humidified air. After 4 h of incubation, the inseminated oocytes were rinsed with G1 Plus medium (Vitrolife, Vastra Frolunda, Sweden). The generated fertilized oocytes were used for electroporation. Zygotes were rinsed with Opti-MEM medium (Thermo Fisher Scientific) and then placed 1-mm electroporation cuvette (P/N 45-0124, Harvard Apparatus, Holliston, USA) filled with 20 μl of Opti-MEM solution containing 600ng/μl Cas9 protein and 600ng/μl gRNA, or 600 ng/μl gRNA alone as mock injection. Electroporation was performed in a Square Wave Electroporation system (BTX, Harvard Apparatus, Holliston, USA) using 30 V, with 1-ms pulse duration and eight pulses separated by 100-ms pulse interval. The zygotes were rinsed with M2 medium (CytoSpring LLC, Mountain View, USA) and cultured in G1 Plus medium (Vitrolife) at 37°C in 5% CO_2_ and 5% O_2_ with humidified air overnight. Surviving two-cell embryos were transferred to the oviducts of pseudo-pregnant Swiss Webster female mice. From the resulting mice, tail and tissue biopsies were collected at postnatal day 7 (P7) and at 2 months of age, then the *C9orf72* G_4_C_2_ repeat length in those samples was analyzed.

### Polymerase chain reaction (PCR)

All the primers used in this study are listed in Supplementary Table S2. (i) To analyze the repeat locus via agarose gel electrophoresis, a 2-primer gene-specific PCR, using primers C9-F1 and C9-R1, was carried out using AmplideX PCR/CE *C9orf72* kit following manufacturer's instructions (Asuragen, Inc, Austin, USA) (64). In the current study, these 2-primer PCR products were referred as ‘2-primer gene-specific PCR products’ to differentiate them from the 3-primer repeat-primed (RP) PCR products as described below. (ii) To analyze the precise base composition at the repeat locus via Sanger sequencing, we used the 2-primer PCR but with primers C9-F2, and C9-R2. Alternative forward primer C9-F3 located 5′ upstream of C9-F2 (sequence not disclosed) was used to analyze the PCR products when C9-F2 did not resolve the sequence. These reactions were carried out following the protocol established by Cleary *et al.* (65) with modifications including the use of 0.8 mM dGTP/dCTP and DreamTaq DNA Polymerase (Thermo Fisher Scientific). (iii) To analyze the Cas9 cleavage site in alleles carrying >150 copies of G_4_C_2_ repeat, we created a 2-primer RP-PCR. In this PCR reaction, one of the primer pair was designed to anneal within the G_4_C_2_ repeat; C9-F5 and C9-R3 primer pair was used to analyze the 5′-DSB (or 5′-SSB) WT Cas9 (or Cas9 nickase) cleavage site. C9-F6 and C9-R4 primer pair was used to analyze the 3′-DSB Cas9 cleavage site. This PCR was performed following the protocol established by Cleary et al. as described in (ii). (iv) To analyze the G_4_C_2_ repeat copy number below 150 copies, we used 3-primer RP-PCR with capillary electrophoresis (CE) resolution. Three primers, C9-F1, C9-R1 and C9-F4, were used in a single reaction. The C9-R1 reverse primer was FAM-labelled to allow for analysis of the RP-PCR amplicons via CE. Typical 3-primer PCR products consist of ‘gene-specific PCR products’ amplified by primers that anneal to the specific sequences outside of the G_4_C_2_ repeat (C9-F1 and C9-R1), as well as multiple ‘RP-PCR products’ amplified by the forward internal repeat-primer C9-F4 and the FAM-labelled reverse primer C9-R1 as indicated in Figure 3D, parental panel. This PCR reaction was carried out using AmplideX PCR/CE *C9orf72* kit following manufacturer's instructions. Resulting data was analyzed using GeneMapper software (Thermo Fisher Scientific).

(v) To analyze the *Tcf4* CTG repeats, the corresponding region was amplified using primers located outside of the repeat (Tcf4-F1 and Tcf4-R1) following a modified LA Taq Protocol using 15 μM of each oligo. Samples were characterized using agarose gel electrophoresis and GC-rich template Sanger sequencing. (vi) To analyze *Fxn* GAA repeats, the corresponding region was amplified using primers located outside of the repeat (Fxn-F1 and Fxn-R1) and following a published protocol (66). Samples were characterized using agarose gel electrophoresis and STRique as described below.

### Sanger sequencing

For PCR products that contained fewer than 50 copies of the *C9orf72* G_4_C_2_ repeats and the *Tcf4* CTG repeats, sequencing was completed using a standard GC-rich Sanger sequencing protocol. Briefly, PCR products were purified with Qiaquick PCR purification kit (Qiagen, Hilden, Germany) and sequenced on an 3730xl DNA Analyzer (Thermo Fisher Scientific), which was ran for 50 PCR cycles after the addition of 3.0 μl 5M betaine (Sigma-Aldrich) and 1.5 ul PCRX Enhancer (Thermo Fisher Scientific). For the 5′-DSB/SSB samples, the amplicons were purified, and Sanger sequenced using C9-F5 as a sequencing primer. 3′-DSB samples were processed similarly, and were Sanger sequenced using C9-R4 as a primer. For PCR products that contained larger than 50 copies of G_4_C_2_ repeats, samples were sequenced using a modified protocol for high GC-content. Briefly, after the column purification, samples were prepared for sequencing using Hairpin DNA & GC-Rich Sequencing pre-mix (MCLAB, South San Francisco, USA) per the manufacturer's instructions and sequenced on an 3730xl DNA Analyzer. BigDye Terminator v3.1 (Thermo Fisher Scientific) and PCRX Enhancer were added to all samples, as well as additional Hairpin DNA & GC-Rich Sequencing premix after denaturation. All Sanger sequencing results were analyzed using Sequencher software (Gene Codes Corporation, Ann Arbor, USA).

### CE G_4_C_2_ repeat length analysis

To measure G_4_C_2_ repeat length, 3-primer RP-PCR amplicons were resolved via capillary electrophoresis (CE) using an 3730xl DNA Analyzer (Applied Biosystems) and Rox1000 ladder (Gel Company Inc., San Francisco, USA) following manufacturers’ instructions. Resulting data was analyzed using GeneMapper software (Thermo Fisher Scientific). For the 5′-DSB/SSB samples, the repeat length was calculated from the size of the most abundant gene-specific PCR product. Since the PCR products have close to 100% GC content, they move through agarose gels differently from the standard size markers. Therefore, following the manufacturer's instruction, calculated values were adjusted using a migration factor which was determine using samples with known G_4_C_2_ repeat length. The repeat length was calculated as follows: (G_4_C_2_ repeat length) = {(the most abundant gene-specific PCR product size determined by capillary electrophoresis)/(migration factor 0.95) – (non-G_4_C_2_ sequence size 175bp)}/6. For 3′-DSB samples, the repeat length was calculated using the size difference between the most abundant gene-specific PCR product and the smallest repeat-primed PCR product as follows: (G_4_C_2_ repeat length) = [{(the most abundant gene-specific PCR product size determined by capillary electrophoresis) – (the smallest repeat-primed PCR product size determined by capillary electrophoresis)}/(migration factor 0.95) – (non-G_4_C_2_ sequence size 175 bp)]/6. For visualizing CE traces, the highest fluorescent signal within indicated size range was defined as 1, and relative signal intensity was shown. The amplicon size indicated in the CE electropherograms was not adjusted by migration factor. To quantify minor peaks in *C9orf72*^hu96x/+^*; Msh2*^+/+^ and *C9orf72*^hu96x/+^*; Msh2*^−/−^ tissues, the number of peaks in CE traces corresponding to the amplicons smaller and larger than that of the highest peak was counted. The signal <20% of the highest signal intensity peak was considered as background and removed from quantification. The repeat length of samples that had >150 copies of G_4_C_2_ repeats was determined by Southern blotting and STRique analysis using nanopore sequencing reads. *Tcf4* CTG repeat length was determined by Sanger sequencing results. The expanded *Fxn* GAA repeat length was determined by STRique analysis using nanopore sequencing reads as described below.

### Repeat instability analysis

Following criteria was used to determine the consequence of repeat instability. For the *C9orf72*^hu96x/+^ mES cell line, those clones that had 105 copies of G_4_C_2_ repeats (more than additional 10 copies G_4_C_2_ repeats) and above were defined as ‘expanded’ clones. Those clones that had 90–104 copies of G_4_C_2_ were defined as ‘retained’ clones. Those clones that had fewer than 90 copies of G_4_C_2_ were defined as ‘contracted’ clones. The rearranged clones that lost 5′ PCR priming site corresponding to C9-F1 sequence, or 3′ PCR priming site corresponding to C9-R1 sequence, were defined as ‘large deletion’ clones. In those samples with mosaicism, each G_4_C_2_ repeat species was counted as an independent allele. For *Tcf4*^hu60x/+^ mES cell line, the 2-primer PCR amplicons larger and smaller than the parental (CTG)_60_-containing amplicon on agarose gels were preliminary called ‘expanded’ and ‘contracted’ clones respectively. The repeat expansions and contractions were confirmed by Sanger sequencing. In those clones with mosaicism, each CTG repeat species was counted as an independent allele. For the *Fxn^hu400x/+^* mES cell line, 2-primer PCR amplicons larger than the control (GAA)_400_-containing amplicon on agarose gels were preliminary called ‘expanded’ and ‘contracted’ clones respectively. The repeat expansions were confirmed by STRique analysis using nanopore sequencing reads. In those clones with mosaicism, each GAA repeat species was counted as an independent allele.

For intergenerational repeat instability, the G_4_C_2_ repeat length was estimated by the mobility on agarose gels visualized by Southern blotting except 96× G_4_C_2_ line. The copy number in 96× G_4_C_2_ line was determined by 3-primer RP-PCR. The consequence of the repeat instability was defined as follows. Expansion, the repeat length 1.5-fold or more compared to the parental one. Contraction, the repeat length 0.5-fold or less compared to the parental one. Retention, the repeat length is in between 0.5-fold and 1.5-fold compared to the parental one.

### Nanopore sequencing/STRique analysis

To count the copy number of the G_4_C_2_ repeats in the targeting vector, the DNA library was prepared from 400 ng of the targeting vector using Rapid Sequencing Kit (SQK-RAD004, Oxford Nanopore Technology, Oxford, UK) following the manufacturer's protocol. The sequencing was performed using a Flo-Min106 flowcell on GridION (Oxford Nanopore Technology). The G_4_C_2_ repeat copies were counted by STRique program (67) using the nanopore sequencing reads. 150 bp immediately upstream and downstream of the G_4_C_2_ repeats were used as Prefix and Suffix sequences respectively. Score (≥4.0) for both Prefix and Suffix was used. To count the copy number of the G_4_C_2_ repeats in mES cells, genomic DNA from following ES cells were collected; *C9orf72*^hu96x/+^, *C9orf72*^hu250x/+^, *C9orf72*^hu300x/+^, *C9orf72*^hu400x/+^, *C9orf72*^hu450x/+^ and *C9orf72*^hu550x/+^ ES cells. The libraries were prepared from genomic DNA samples (5–10 μg each) using Cas9 Sequencing kit (SQK-CS9109, Oxford Nanopore Technology), WT Cas9 (IDT), and gRNAs (C9-ONT-1, C9-ONT-2, C9-ONT-3, and C9-ONT-4 in Supplementary Table S1). The sequencing was performed using a Flo-Min106D flow cell on GridION, and STRique program with the same criteria above was used to count the copy number of the G_4_C_2_ repeats in each clone. The copy number of GAA repeats was counted as follows. The *FXN* GAA repeats in humanized *Fxn* ES cell clones were amplified by PCR. Amplicons were purified and ONT sequencing adaptors were ligated using Ligation Sequencing Kit. The sequencing was performed using a Flo-Min106D flow cell on GridION. STRique program was used to count the copy number of the GAA repeats. 150 bp immediately upstream and downstream of the GAA repeats were used as Prefix and Suffix sequences respectively. Score (≥4.0) for both Prefix and Suffix was used.

### Southern blotting

Southern blotting was performed following a standard protocol. Briefly, 5–10 μg of total genomic DNA extracted from mES cells or mouse tissues were digested with SspI and XhoI. The digested genomic DNA fragments were separated in 1% agarose gel, transferred to Hybond-N+ hybridization membrane (Sigma-Aldrich), and then hybridized with P^32^-labeled probe that contained mouse (Chr 4: 35226703–35226428) and human (Chr 9: 27573563–27573740) sequences. The probe was designed to recognize both mouse WT and humanized *C9orf72* as seen in Figure 8C. After washing the membrane with SSC buffer, the P^32^ signal was visualized using Phosphor imager (Perkin Elmer, Waltham, USA).

### Cell culture

Mouse ES cells were cultured in DMEM containing 15% FBS, 100 units/ml of penicillin, 100 μg/ml of streptomycin, 1× non-essential amino acid, 1× GlutaMax and 0.1 mM 2-mercaptoethanol, and 2000 U/ml LIF (all from Thermo Fisher Scientific) with following modifications. 3000 and 2200 mg/l of NaCl and NaHCO_3_ were used in DMEM to adjust osmolality to 200 mOsm/kg from original 340 mOsm/kg. Passage numbers refer to the passage after each gene editing/targeting electroporation. When additional genome modification was made, the passage number was reset to passage 1 again. For H_2_O_2_ treatment, cells were trypsinized and incubated with vehicle or 5 mM H_2_O_2_ (Sigma-Aldrich) in culture media for 15 min, neutralized by washing with culture media twice and plated on feeder layer on day 1. On day 5, cells were replated on gelatin coated plates and colonies were isolated 10 days later. To characterize the *C9orf72* G_4_C_2_ repeats over passages, frozen mES cells were thawed, cultured 48 h per passage on cell culture plates. Genomic DNA was collected after trypsinization and neutralization. For DSB/SSB introduction experiments, unless otherwise noted, frozen mES cells were thawed and plated on day 1, replated on day 3, electroporated after trypsinization and plated on feeder cells on day 5. On day 7, cells were trypsinized and replated on gelatin-coated plates. Colonies were isolated 10 days after the electroporation. Electroporation was performed by 4D-Nucleofector X Unit in 100 μl Nucleocuvette Vessel on program CP-105 (LONZA, Basel, Switzerland). The subclones were grown in 96 well plate until confluency for downstream processes. For subcloning experiments, the same procedure was applied except that these cells did not receive electroporation. For the introduction of DSBs or SSBs, 122 pmol of WT Cas9, or Cas9-D10A nickase, was pre-incubated with 230 pmol of sgRNA (IDT), and electroporated into 2 × 10^6^ cells on day 5 in our standard protocol described above. For siRNA treatment, frozen mES cells were thawed and plated on day 1, replated on day 3, 30 pmol of *Rad51* siRNA, 100 pmol of *Pold3* siRNA, or control siRNA (30 pmol for *Rad51* siRNA experiments and 100 pmol for *Pold3* siRNA experiments) was electroporated into 2 × 10^6^ cells and plated on feeder layer on day 5, Cas9 and gRNA with *Rad51* siRNA or with control siRNA were electroporated and plated on feeder layer on day 6. Cells were replated on gelatine coated plates on day 7. Colonies were isolated 10 days after the last electroporation. The subclones were cultured in 96 well plate until confluency. For knockdown experiments, WT Cas9 and gRNA were introduced into *C9orf72*^hu96x/+^ that received either *Rad51* siRNA, *Pold3* siRNA, or control scramble siRNA. Alternatively, Cas9-D10A nickase and gRNA were introduced into *C9orf72*^hu96x/+^ that received either *Rad51* siRNA, *Pold3* siRNA, or control scramble siRNA. Frequencies of the indicated types of rearrangement was calculated from each experiment.

### Statistical analyses

Student's *t*-test was performed (paired, two-tailed) to analyze the *Rad51* knockdown, *Pold3* knockdown, or *Pif1* KO experiments.

### Western blotting

Cells were lysed in RIPA buffer containing Halt™ proteinase inhibitor cocktail (both from Thermo Fisher Scientific). 300 ng of total lysates were subjected to western blotting using Wes (ProteinSimple), using 12–230 kDa separation module following the manufacturer's instruction. Anti-Msh2, anti-Rad51, anti-Pold3 and anti-b-actin were used as 1:25 dilution. The data was analyzed using Compass for SW software (Protein Simple).

### Quantitative PCR

For analyzing transcript level, total RNA was extracted with Zymo Quick RNA 96 kit (Zymo Research, Irvine, CA) and samples were adjusted to 10 ng/ml. Gene expression analysis was performed by multiplex TaqMan qRT-PCR using QuantiNova RT-PCR kit (Qiagen) in 384-well PCR plates run on QuantStudio thermocyclers (Thermo Fisher). Probes for *Pold3* (Mm00713051_m1) and *Drosha* (Mm01310009_m1) were purchased from Thermo Fisher. Gene KO in mES cells were confirmed by Taqman qPCR as previously described (57). Probes and primer sequences for *Msh2* KO and *Pif1* KO are listed in Supplementary Table S3.

## Results

### Generation of humanized *C9orf72* alleles

Several mouse models have been created to study ALS/FTD associated with the *C9orf72* G_4_C_2_ repeat expansion (49–52). These transgenic mice were generated using BACs derived from human patient DNA. Since these BACs were inserted into the mouse genome by random integration, these transgenes may be influenced by chromosomal position effects, and the resulting phenotype may vary among lines. To avoid these concerns, we generated knock-in alleles at the endogenous mouse *C9orf72* locus. The human *C9orf72* gene has two transcription start sites (TSSs) located in two distinct exons, referred to as exon 1a and exon 1b, and the G_4_C_2_ repeats are located in between these two exons (Figure 1A). Since mouse *C9orf72* does not have a G_4_C_2_ repeat at the corresponding intron, we replaced this intron and its surrounding sequences, a part of exon1a, exon1b, and a part of the intron downstream of exon1b, with the human counterpart (humanized *C9orf72* allele) via homologous recombination in mES cells (57). We successfully inserted 96 copies of G_4_C_2_ repeats (*C9orf72*^hu96x/+^) that is three-fold larger than the disease threshold (Figure 1B). As a control allele, we inserted 3 copies of G_4_C_2_ repeats (*C9orf72*^hu3x/+^). (See detailed alleles description in Materials and Methods).

### *C9orf72* G_4_C_2_ repeat instability *in vitro* and *in vivo*

First, we asked if G_4_C_2_ repeats in the humanized allele exhibit instability in targeted mES cells. In contrast to the stable G_4_C_2_ repeats in *C9orf72*^hu3x/+^ mES cells, the copy number of the most abundant G_4_C_2_ repeat species (referred to as repeat length of a given sample hereinafter) in *C9orf72*^hu96x/+^ mES cells slowly increased during *in vitro* passage (Figure 1C), as measured by repeat-primed PCR (RP-PCR) followed by capillary electrophoresis (CE; see Materials and Methods for detailed description). Indeed, the repeat length in the *C9orf72*^hu96x/+^ mES cell line at passage 4 after initial targeting was already larger than the targeting vector and continued to expand over passages. The modest increase in repeat length was also confirmed after a single subcloning in *C9orf72*^hu96x/+^ mES cells but not in *C9orf72*^hu3x/+^ mES cells (Figure 1D). These results demonstrate a greater degree of repeat instability in the pathogenic length *C9orf72* G_4_C_2_ repeats compared to the control.

**Figure 2.**
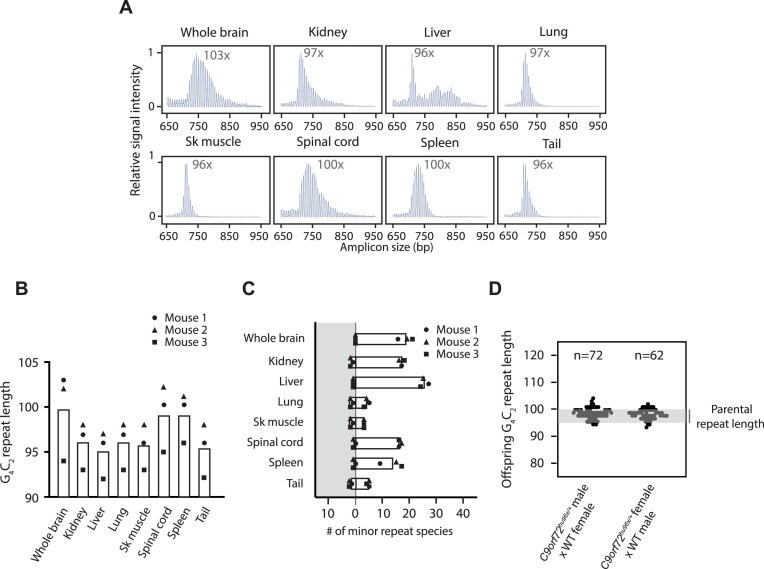
Somatic and intergenerational repeat instability in *C9orf72^hu96/+^* mice. (**A**) Representative 3-primer RP-PCR CE traces from 6 months-old *C9orf72*^hu96x/+^ tissues. The G_4_C_2_ repeat length (the G_4_C_2_ repeat copy number corresponding to the highest peak in the CE traces) is indicated in the graphs. Sk muscle: skeletal muscle. (**B**) The G_4_C_2_ repeat length in tissues. Each circle, triangle and square represents an individual sample. White bars indicate mean values. *n* = 3 mice analyzed. (**C**) Quantification of the minor G_4_C_2_ repeat species. The number of peaks larger (white background), and smaller (grey background) than the highest peak in indicated tissues is shown. Each circle, triangle, and square represents an individual sample. White bars indicate mean values. *n* = 3 mice analyzed. (**D**) Intergenerational repeat instability. The repeat length in the offspring from *C9orf72*^hu96x/+^ heterozygous mice bred with WT. *n* = 72 and *n* = 62 mice analyzed from paternal and maternal inheritance respectively.

We subsequently generated mice from *C9orf72*^hu96x/+^ mES cells. Consistent with the observation in cultured mES cells, *C9orf72*^hu96x/+^ mice accumulated additional copies of G_4_C_2_ repeats in their tissues by 6 months of age, showing somatic repeat instability (Figure 2A–C). As seen in other RED models, the degree of instability was tissue type-dependent (5,23,25,28,32,68). The G_4_C_2_ repeats in tissues such as tail or skeletal muscle were relatively stable, enabling us to genotype animals using tail biopsies. In some tissues, such as whole brain, spinal cord or spleen, the highest peak in CE traces shifted toward a larger size and the distribution of the peaks became broader, showing an overall trend toward repeat expansion. In other tissues such as liver, the number of minor peaks increased dramatically and exhibited high heterogeneity. These results confirmed the *C9orf72* G_4_C_2_ somatic repeat instability in this model.

We next looked at intergenerational instability in *C9orf72*^hu96x/+^ mice. The G_4_C_2_ repeat length in the tails of P7 neonates derived from *C9orf72*^hu96x/+^ mice (95–97 copies of G_4_C_2_ repeats, intercrossed with *C9orf72*^+/+^ mice) was between 92–103 copies, in the range between 0.95- and 1.06-fold of parental repeat length (Figure 2D). Even though we observed this minor instability, we did not detect large (>1.5-fold increase) intergenerational repeat expansions. We chose this threshold because >1.5-fold change between generations is common among disease-causing STRs but quite rare in other RED mouse models (2,14,19,69,70)

The absence of major repeat instability in our humanized *C9orf72*^hu96x/+^ allele prompted us to further dissect the molecular mechanisms of these apparently distinct types of repeat instability.

### Repeat expansion and contraction triggered by DNA DSB and SSB

**Figure 3.**
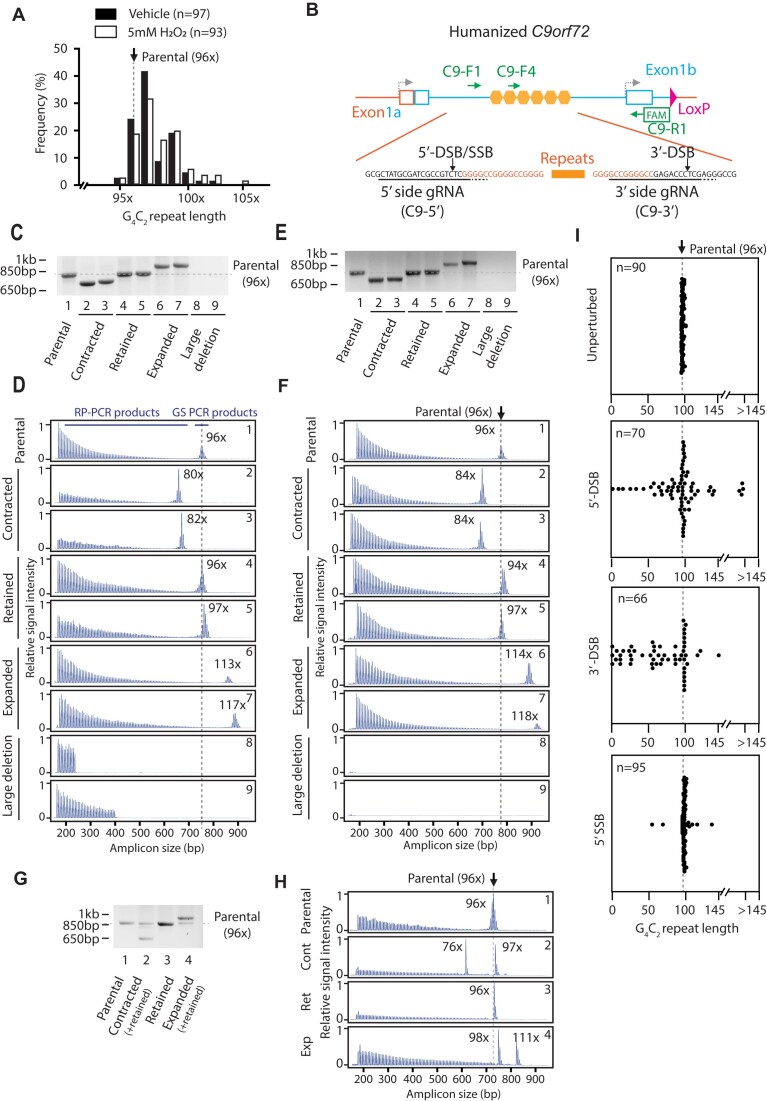
G_4_C_2_ repeat expansions induced by a DNA DSB or SSB in mES cells. (**A**) G_4_C_2_ repeat length changes by H_2_O_2_ treatment. *C9orf72*^hu96x/+^ ES cells were treated with H_2_O_2_ or vehicle for 15 min and the repeat length was analyzed after subcloning. Parental repeat length shown as dotted line. (**B**) DSB/SSB introduction by CRISPR-Cas9 at the *C9orf72* G_4_C_2_ locus. Schematic of the *C9orf72* G_4_C_2_ locus shown on the top. Green arrows indicate PCR primers. C9-R1 reverse primer is FAM-labelled for CE analysis. C9-F4 forward primer serves as a G_4_C_2_ repeat-priming for RP-PCR. C9-F1/C9-R1 and C9-F1/C9-F4/C9-R1 were used for 2-primer gene-specific PCR and 3-primer RP-PCR respectively. Cas9 guide sequences and cleavage sites are shown in the bottom. (**C**) Analysis of the G_4_C_2_ repeat locus by 2-primer gene-specific PCR after 5′-DSB introduction in mES cells. Gel electrophoresis of amplicons from representative clones shown. A dotted line indicates the G_4_C_2_ repeat length in the parental clone (96 copies of G_4_C_2_ repeats). (**D**) 3-primer RP-PCR CE traces from representative clones after 5′-DSB introduction in mES cells. The numbers at top right corner correspond to the lane number in (C). The G_4_C_2_ repeat length corresponding to the highest CE peak is indicated in the panels. A dotted line indicates the G_4_C_2_ repeat length in the parental clone (96 copies of G_4_C_2_ repeats). In the top parental panel, RP-PCR products derived from repeat-primed reaction (C9-F4/C9-R1 reaction in B) and gene-specific (GS) PCR products derived from *C9orf72* gene sequence-specific reaction (C9-F1/C9-R1) are indicated. (**E**) Analysis of the G_4_C_2_ repeat locus by 2-primer gene-specific PCR after 3′-DSB introduction in mES cells. (**F**) 3-primer RP-PCR CE traces from representative clones after 3′-DSB introduction in mES cells. The numbers at top right corner correspond to the lane number in (E). (**G**) Analysis of the G_4_C_2_ repeat locus by 2-primer gene-specific PCR after 5′-SSB introduction in mES cells. (**H**) 3-primer RP-PCR CE traces from representative clones after 5′-SSB introduction in mES cells. The numbers at top right corner correspond to the lane number in (G). Cont: contracted; Ret: retained; Exp: expanded. (**I**) The G_4_C_2_ repeat length after introducing indicated DNA lesions in mES cells. Representative data from a 96-well plate analysis shown. Dotted lines indicate the G_4_C_2_ repeat length in the parental clone (96 copies of G_4_C_2_ repeats). Due to the assay detection limit, those clones with >145 copies of G_4_C_2_ repeats were grouped in the graphs.

To gain insight into major repeat instability, we looked for factors that can induce large-scale repeat expansions in the mouse genome. Since DNA damage is a known factor affecting genome stability (71,72), we tested if global DNA damage, induced by H_2_O_2_, could trigger large repeat expansions at our humanized G_4_C_2_ repeat locus in cultured mES cells. H_2_O_2_ treatment of *C9orf72*^hu96x/+^ cells resulted in a minor shift in G_4_C_2_ repeat length distribution after subcloning: up to nine additional copies of G_4_C_2_ by H_2_O_2_ treatment, a slight increase from up to seven additional copies by vehicle-treatment (Figure 3A).

We speculated that introducing DNA DSBs specifically at the *C9orf72* G_4_C_2_ repeats could have a greater impact on repeat instability rather than stochastic genome-wide H_2_O_2_-induced damage. We therefore took advantage of the specificity of the CRISPR-Cas9 system to introduce a DSB adjacent to the 5′ end of the G_4_C_2_ repeats in *C9orf72^hu96x/+^* mES cells (Figure 3B), followed by subcloning and repeat length analysis (via 2-primer PCR and 3-primer RP-PCR, and respective agarose and CE visualization). 81.5% of subclones yielded 2-primer gene-specific PCR products. Among these, 18.3% showed a significant increase in repeat length (more than 10 copies of G_4_C_2_ added, examples shown in Figure 3C lanes 6 and 7, Figure 3D ‘Expanded’ panels, and summarized in Table 1) compared with the parental clone. This increase exceeded the largest spontaneous shift (7 copies of G_4_C_2_ added) observed after a single subcloning (Figure 1D). Subclones in this category had non-interrupted G_4_C_2_ repeats when analyzed by Sanger sequencing (examples shown in Supplementary Figure S3). 42.7% of subclones had a similar length of the repeats to the parental clone (90–104 copies of G_4_C_2_, examples shown in Figure 3C, lanes 4 and 5, Figure 3D, ‘retained’ panels), and 21.8% of subclones contained contracted G_4_C_2_ repeats (fewer than 90 copies of G_4_C_2_, examples shown in Figure 3C lanes 2 and 3, Figure 3D, ‘contracted’ panels). 18.5% of subclones did not yield a 2-primer gene-specific PCR product (examples shown in Figure 3C, lanes 8 and 9), but they did show RP-PCR products (Figure 3D, large deletion panels), suggesting the 5′ primer binding site was deleted by end-processing at the site of the DNA DSB.

**Table 1.** Analysis of the repeat instability in mES cells

Gene	*C9orf72*	*C9orf72*	*C9orf72*	*C9orf72*	*C9orf72*	*C9orf72*	*C9orf72*	*C9orf72*	*Tcf4*	*Tcf4*	*Fxn*	*Fxn*
STR sequence	G_4_C_2_	G_4_C_2_	G_4_C_2_	G_4_C_2_	G_4_C_2_	G_4_C_2_	G_4_C_2_	G_4_C_2_	CTG	CTG	GAA	GAA
Additional modification	None	None	None	None	None	None	*Msh2^−/−^*	*Msh2^−/−^*	None	None	None	None
gRNA location	5′	5′	5′	3′	3′	3′	5′	5′	5′	5′	3′	3′
Parental repeat length	31×	96×	96×	31×	96×	250×	97×	97×	60×	60×	400×	400×
Cas9	WT	WT	Nick^D10A^	WT	WT	WT	WT	Nick^D10A^	WT	Nick^D10A^	WT	Nick^D10A^
# of samples analyzed	100	250	300	97	89	113	195	106	107	195	89	188
Large deletion (%)	32.0	18.5	0	22.7	27.0	3.5	15.9	0	0.9*	0	4.5*	0
Contraction (%)	11.0	21.8	1.3	55.7	40.4	40.7	22.6	0.9	8.4	4.1	0	0
Retention (%)	51.0	42.7	92.3	21.6	29.2	42.5	46.2	92.5	84.1	95.9	92.1	100
Expansion (%)	6.0	18.3	6.3	0	3.4	13.3	15.4	6.6	6.5	0	3.4	0
Expansion/contraction ratio	0.55	0.84	4.85	0	0.08	0.33	0.68	7.33	0.78	NA	NA	NA
Expansion/retention ratio	0.12	0.41	0.07	0	0.12	0.31	0.33	0.07	0.08	0	0.04	0
Contraction/retention ratio	0.22	0.51	0.01	2.58	1.38	0.96	0.49	0.01	0.10	0.04	0	0
Max repeat length	53×	250×	132×	NA	145×	550×	>145×	>145×	87×	NA	639×	NA
Indel (%) [# of sample sequenced]	96.4 [28]	87.1 [31]	0 [31]	94.4 [25]	96.0 [18]	ND	ND	ND	ND	ND	ND	NA

Nick^D10A^: Cas9 nickase-D10A. NA: not applicable. ND: not determined.

*No amplification by PCR. No detailed deletion analysis performed.

To determine whether the expansion was specific to a DSB at the 5′ end of the repeats, we conducted the same experiment using C9-3′ gRNA, which generates a DSB adjacent to the 3′ end of the G_4_C_2_ repeats, in *C9orf72^hu96x/+^* mES cells (Figure 3B). We observed the same types of expansions and contractions following a 3′-DSB as we did with a 5′-DSB (Figure 3E and F). Since C9-R1 reverse primer was FAM-labelled for CE, unlike the 5′-DSB results we did not detect any signal by 3-primer RP-PCR when 2-primer PCR did not yield any amplicons (Figure 3E, lanes 8 and 9, and Figure 3F, large deletion panels). Sanger sequencing of the cleavage sites in individual clones revealed characteristic small insertions and/or deletions (indels) in >85% of clones, regardless of the type of the repeat alteration (Table 1).

In our efforts to generate repeat-expanded humanized *C9orf72* allele in mES cells, in addition to the *C9orf72*^hu96x/+^ mES cell clone, we also obtained a clone with 31 copies of repeats (*C9orf72*^hu31x/+^ cells, Supplementary Figures S3C and S4B). After confirming that these 31 copies of G_4_C_2_ repeats were stable over several passages in mES cells (Supplementary Figure S4C), we tested the impact of DSB in this clone to determine if starting repeat length affects rearrangement outcome. When a 5′- or 3′-DSB was introduced into *C9orf72*^hu31x/+^ cells, we observed the same types of rearrangements as seen in *C9orf72^hu96x/+^* cells except that 3′-DSB failed to expand the repeats (Table 1). We observed that larger repeats expanded more frequently; 31× (6.0%), 96× (18.3%) by 5′-DSB, as well as 31× (0%) and 96× (3.4%) by 3′-DSB. On the other hand, we did not find a clear trend for contractions. Collectively, the expansions/contractions ratio positively correlated with starting repeat length (31× [0.55] and 96× [0.84] by 5′-DSB and 31× [0] and 96× [0.08], by 3′-DSB), suggesting that starting repeat length impacts the direction of change as either expansions or contractions.

While DSBs clearly had a pronounced effect on G_4_C_2_ repeat length in cultured ES cells, single strand breaks (SSBs) occur far more frequently in mammalian cells (73) and are therefore more physiologically relevant to RED patient alleles. To determine whether a SSB can also cause repeat expansions, we introduced a 5′-SSB into *C9orf72*^hu96x/+^ ES cells using Cas9-D10A nickase and C9-5′ gRNA. A 5′-SSB created by this nickase resulted in both expansions and contractions of the repeats in *C9orf72*^hu96x/+^ cells (Figure 3G and H), but SSB-induced repeat instability exhibited some different characteristics. For example, SSB did not generate any indels regardless of the repeat length alterations (Table 1 and examples shown in Supplementary Figure S4A). Accordingly, none of the subclones showed large deletion mutations (Table 1). Following nickase treatment, many of the rearranged clones exhibited a higher frequency of mosaicism compared with WT Cas9-treated clones (Figure 3G and H). Another difference was the expansions/contractions ratio. While 5′-DSB into 96 copies of G_4_C_2_ repeats generated more contractions than expansions (expansion/contraction ratio was 0.84, Table 1), 5′-SSB generated more expansions than contractions (expansion/contraction ratio was 4.85, Table 1). Despite these differences, both DSB and SSB were capable of inducing expansion of *C9orf72* repeats.

### Repeat expansion by DSB and SSB at other disease-causing STRs

Since DSB introduction on either the 5′ or 3′ side of *C9orf72* G_4_C_2_ repeats could induce repeat expansions, we asked if introducing DSB at other disease-causing STRs could also trigger large repeat expansions. To answer this, we created two novel RED models in which we humanized the respective murine gene and inserted pathogenic length repeats.

**Figure 4.**
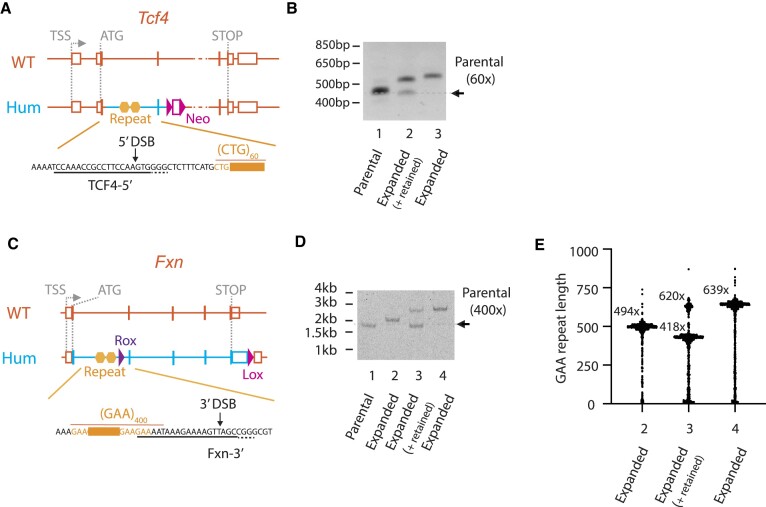
Repeat expansions by a DNA DSB at the *Tcf4* CTG repeats and the *Fxn* GAA repeats in mES cells. (**A**) Humanized *Tcf4* allele schematic. gRNA sequence and Cas9 cleavage site are shown in the bottom. (**B**) The *Tcf4* CTG repeat expansions by 5′-DSB in mES cells. 2-primer gene-specific PCR amplicons from two largest repeat expanded clones shown. A dotted line indicates parental repeat length (60 copies of CTG). (**C**) Humanized *Fxn* allele schematic. gRNA sequence and Cas9 cleavage site are shown in the bottom. (**D**) The *Fxn* GAA repeat expansions by 3′-DSB in mES cells. 2-primer gene-specific PCR amplicons from repeat expanded clones shown. A dotted line indicates parental repeat length (400 copies of GAA). (**E**) Nanopore sequencing/STRique repeat analysis. Most frequently called repeat length(s) shown as horizontal bars and numbers. The numbers on X-axis correspond to the lane numbers in (D). The number of nanopore sequence reads used for STRique analysis were, 2 (*n* = 763), 3 (*n* = 693) and 4 (*n* = 899), respectively. Orange: mouse; blue: human; yellow hexagons: gene-specific repeats. TSS: transcription start site.

Fuchs’ corneal dystrophy (FCD) is an eye disease that causes gradual vision loss due to the disfunction of corneal endothelial cells. The most common cause of FCD is CTG triplet repeat expansion in an intron of *TCF4* (74,75). We humanized the *Tcf4* allele by gene targeting in mouse ES cells using a BAC-based targeting vector that replaced mouse *Tcf4* intron 2, exon 3 and part of intron 3 with the human counterpart including pathogenic 60 copies of CTG repeats (Figure 4A, Supplementary Figure S5A, and Materials and Methods). We designed a gRNA (TCF4-5′) to help induce a DSB 14 bp upstream of the 5′ end of the CTG repeats when delivered together with WT Cas9. This gRNA was electroporated into *Tcf4*^hu60x/+^ mES cells with Cas9 protein and the CTG repeat length of resulting subclones were analyzed. Screening of subclones identified repeat-expanded (6.5%) as well as repeat-contracted subclones (8.4%), as seen in humanized *C9orf72* G_4_C_2_ repeats. The expansion/contraction ratio in this clone was 0.78. The *Tcf4* CTG repeats appeared more stable than *C9orf72* repeats after 5′-DSB as 84.1% of subclones retained 60× repeats, compared to 42.7% and 51.0% in *C9orf72*^hu96x/+^ and *C9orf72*^hu31x/+^ subclones respectively after 5′-DSB. The two most expanded subclones are shown in Figure 4B, lanes 2 and 3. Sanger sequencing of these amplicons confirmed that the two expanded subclones obtained additional 16 and 17 copies of CTG, resulting in 86 and 87 copies of CTG, respectively (Supplementary Figure S6). We also tested the impact of a 5′-SSB by electroporating Cas9-D10A nickase and TCF4-5′ gRNA. Unlike the case of 96 copies of *C9orf72* G_4_C_2_ repeats, 5′-SSB did not induce repeat expansions whereas repeat contractions were still observed (4.1%, see Table 1).

We then examined a third STR, GAA triplet repeats in *FXN* intron 1 that is known to cause FA, the most frequent hereditary ataxia (76). A novel repeat-expanded, humanized *Fxn* allele was generated by two successive gene targeting events in mES cells. First, the entire mouse *Fxn* coding sequences and introns were replaced by those of human *FXN* using a BAC-based targeting vector (see scheme in Supplementary Figure S5Bi–v). A second targeting vector which contained 650 copies of GAA repeats was constructed and electroporated into WT humanized *Fxn* ES cells (Supplementary Figure S5Bvii–ix). 650 copies of GAA repeats in the targeting vector collapsed down to 400 copies during gene targeting. Using this humanized *Fxn* cell line with 400 copies of GAA repeats (Humanized *Fxn*^hu400x/+^ mES cells), we tested for repeat expansions. A gRNA (Fxn-3′) was designed to introduce a DSB 13 bp downstream of GAA repeats (Figure 4C). Introduction of a 3′-DSB using WT Cas9 and the gRNA produced repeat-expanded subclones (3.4%) as identified via 2-primer PCR amplification (Figure 4D). Unexpectedly, we did not identify any repeat-contracted subclones in this experiment. Similar to the case of *Tcf4* CTG repeats, *Fxn* GAA repeats were relatively stable compared to *C9orf72* G_4_C_2_ repeats, as the vast majority of subclones (92.1%) retained the repeat length after a DSB introduction. Further characterization of repeat-expanded subclones using nanopore sequencing and STRique analysis (67) showed this manipulation added 100–240 copies of GAA repeats, resulting in 500–640 copies of GAA repeats in these subclones (Figure 4E). When 3′-SSB was introduced into humanized *Fxn*^hu400x/+^ mES cells by Cas9-D10A nickase, we failed to detect any expansions, contractions, or large deletions (Table 1).

### Large repeat expansion resulting from DNA DSB/SSB is HDR-dependent

**Figure 5.**
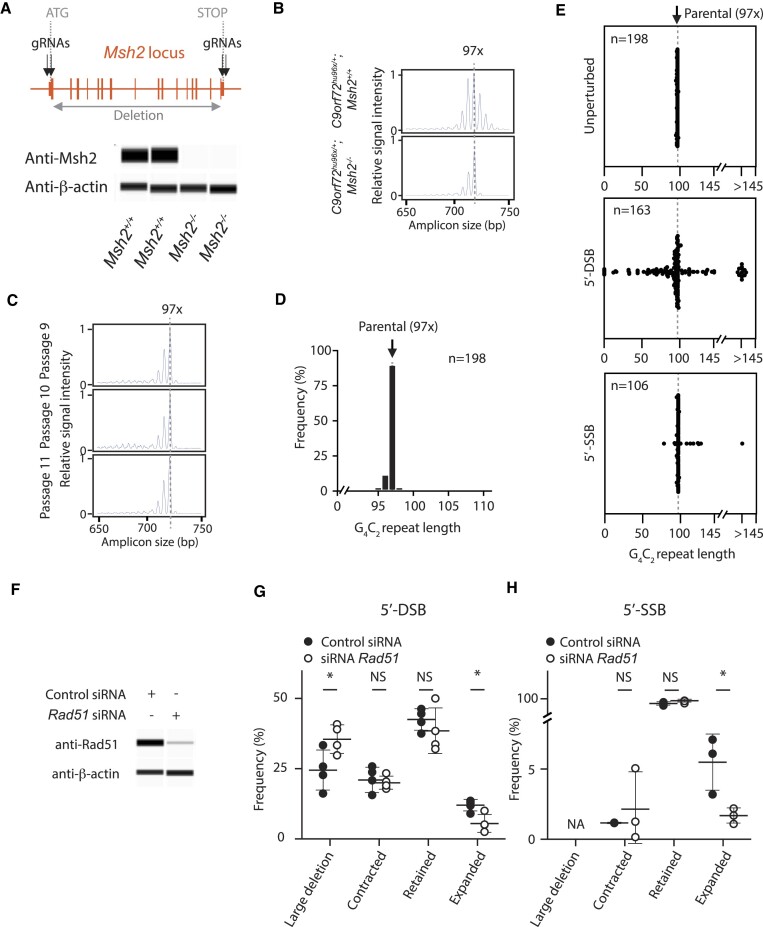
DNA DSB/SSB-induced repeat expansions are HDR-dependent. (**A**) Top, *Msh2* null allele schematic. gRNA locations for gene deletion indicated. Bottom, western blotting analysis to confirm deletion of Msh2 in *C9orf72*^hu96x/+^*; Msh2*^−/−^ ES cells. (**B**) 3-primer RP-PCR CE traces from *C9orf72*^hu96x/+^*; Msh2*^+/+^ and *C9orf72*^hu96x/+^*; Msh2*^−/−^ ES cells. A dotted line indicates the G_4_C_2_ repeat length in the parental clone (97 copies of G_4_C_2_ repeats). (**C**) 3-primer RP-PCR CE traces from *C9orf72*^hu96x/+^*; Msh2*^−/−^ ES cells at indicated passages. A dotted line indicates 97 copies of G_4_C_2_ repeats. (**D**) The G_4_C_2_ repeat length after a single subcloning *C9orf72*^hu96x/+^; *Msh2*^−/−^ ES cells. *n* = 198 subclones analyzed. A dotted line indicates the G_4_C_2_ repeat length in the parental clone (97 copies of G_4_C_2_ repeats). (**E**) The G_4_C_2_ repeat length analysis after indicated DNA lesions in *C9orf72*^hu96x/+^; *Msh2*^−/−^ ES cells. *n* = 198, *n* = 163 and *n* = 106 clones were analyzed in unperturbed condition, or after 5′-DSB and 5′-SSB, respectively. A dotted line indicates the G_4_C_2_ repeat length in the parental clone (97 copies of G_4_C_2_ repeats). Due to the assay detection limit, those clones with >145 copies of G_4_C_2_ repeat were grouped in the graphs. (**F**) Anti-*Rad51* western blotting analysis to confirm siRNA efficiency in *C9orf72*^hu96x/+^ ES cells. (**G**, **H**) Frequencies of the indicated changes after 5′-DSB (G) or 5′-SSB (H) introduction with *Rad51* siRNA in *C9orf72*^hu96x/+^ ES cells. Four (G) and three (H) independent experiments were performed respectively. * *P*< 0.05. NS, not significant. NA, not applicable.

A large number of studies demonstrated that proteins in the DNA MMR pathway, including Msh2, play important roles in somatic repeat instability (2,21,23,24,26,28,29,31,77). Msh2 is a component of the MutS complex that recognizes DNA mismatches and stimulates downstream reactions. Loss of *Msh2* prevents somatic repeat instability in several RED models including HD, DM1 or FXS (2,21,22). Therefore, we deleted the *Msh2* gene in *C9orf72*^hu96x/+^ mES cells to test its contribution to the *C9orf72* G_4_C_2_ repeat instability (Figure 5A and Supplementary Figure S5C, D). When we compared the CE traces between *C9orf72*^hu96x/+^*; Msh2*^+/+^ and *C9orf72*^hu96x/+^*; Msh2*^−/−^ ES cells in the unperturbed condition, minor peaks beyond 97x were no longer observed in *C9orf72*^hu96x/+^*; Msh2^−/−^* ES cells (Figure 5B). In contrast to *C9orf72*^hu96x/+^*; Msh2*^+/+^ mES cells, the repeats in *C9orf72*^hu96x/+^*; Msh2*^−/-^ cells were stable during *in vitro* passage (compare Figures 1C and 5C). The *C9orf72* G_4_C_2_ repeats were also stable after subcloning in *Msh2* null cells (compare Figure 1D and 5D): 1.0% of subclones had one additional copy compared to the parental 97 copies (98 copies of G_4_C_2_). 9.9% of subclones had one copy fewer (96 copies of G_4_C_2_) and 1.0% of subclones had two copies fewer (95 copies of G_4_C_2_). These data confirmed Msh2 contributes to G_4_C_2_ repeat instability in the unperturbed condition. However, when a 5′-DSB was introduced into *C9orf72*^hu96x/+^*; Msh2*^−/−^ ES cells, this DSB introduction led to repeat expansions (>10 additional copies of G_4_C_2_) (Figure 5E), similar to our observations in *C9orf72*^hu96x/+^*; Msh2*^+/+^ cells (compare the distribution pattern in Figure 3I 5′-DSB panel and Figure 5E 5′-DSB panel, and Table 1), indicating that the large-scale repeat expansions associated with a DNA DSB does not require Msh2.

From the observation that a single electroporation of WT Cas9 and gRNA increased G_4_C_2_ repeat length >2-fold (Table 1 and Figure 8C), we speculated that the extra G_4_C_2_ repeats may arise through HDR pathway. To test this hypothesis, we knocked down *Rad51*, a central factor of HDR (Figure 5F), and assayed the frequency of large repeat expansions. As a positive control for the inhibition of HDR by the *Rad51* siRNA, we electroporated aliquots of *Rad51* siRNA- and control siRNA-treated cells with a BAC-based construct targeting *Rosa26*. As expected, *Rad51* knockdown inhibited *Rosa26* targeting (Supplementary Figures S7A and S7B). When a DSB was introduced into *C9orf72*^hu96x/+^ ES cells by WT Cas9 and C9-5′ gRNA, the *Rad51* siRNA treatment significantly reduced the frequency of repeat expansions compared to the control siRNA (Figure 5G). Interestingly, the frequency of the events, repeat retention or contractions, was unaffected by *Rad51* knockdown, whereas that of large deletions was significantly higher.

Since repeat expansions by a SSB occurred also in the absence of Msh2 (Figure 5E, 5′-SSB panel and Table 1), we tested if inhibiting the HDR pathway produces similar results. We delivered *Rad51* siRNA and control siRNA into the *C9orf72*^hu96x/+^ ES cells and introduced a 5′-SSB by Cas9-D10A nickase and C9-5′ gRNA. Similar to the case of WT Cas9, *Rad51* knockdown by siRNA consistently inhibited, but did not abrogate, major repeat expansions induced by a 5′-SSB, whereas the frequency of contractions and retention were not altered (Figure 5H), suggesting the expansions triggered by a DSB and a SSB were both HDR-dependent.

Despite the shared Rad51 dependency, given the different dynamics at broken DNA ends caused by DSB and SSB, we suspected that repeat expansions induced by these lesions can be achieved through different HDR pathways. A DSB generates two DNA ends (two-ended DSB), and when repaired by HDR, this is mainly achieved by error-free HDR pathways, either synthesis-dependent strand-annealing (SDSA) or canonical double-strand break repair (DSBR) with formation of double Holliday junctions (78). On the contrary, a SSB during DNA replication in S phase, unless rescued by a proximal incoming replication fork, can become a one-ended DSB that is repaired by BIR, which is an error-prone HDR pathway (78–80). Hence, we hypothesized that SSB-induced repeat expansions, but not DSB-induced repeat expansions, were mediated by BIR. BIR exhibits substantially higher mutation rates in yeast and human (81,82). Kononenko *et al.* reported elevated levels of mutation associated with *FMR1* CGG repeat instability outside of the repeats, attributed to BIR, in a murine cancer cell line (40). Therefore, we analyzed the sequences near the Cas9-D10A nickase cleavage site in those SSB-induced G_4_C_2_ repeat-expanded clones expecting to detect such mutations associated with BIR, but failed to find any mutation outside of the G_4_C_2_ repeats within approximately 1 kb 5′ upstream or 3′ downstream from the Cas9-D10A cleavage site in those repeat-expanded clones examined (*n* = 6, example shown in Supplementary Figure S10). Though BIR has higher mutation rates, the frequency of mutation by BIR may be far lower than we can capture in mES cells. Therefore, we approached this hypothesis by suppressing *Pold3* or removing *Pif1*, both of which are reportedly key proteins involved in mammalian BIR (40,41,83–85). Knocking down *Pold3* or knocking out *Pif1* in *C9orf72*^hu/96x^ cells did not significantly alter the G_4_C_2_ repeat length distribution in the absence of exogenous DSB/SSB introduction (Supplementary Figures S7C–E and S7H–J). Unexpectedly, even when a 5′-DSB or a 5′-SSB was introduced in *Pold3*-knocked-down *C9orf72*^hu/96x^ cells or *C9orf72*^hu/96x^; *Pif1*^−/−^ cells, we did not observe significant changes in frequencies of repeat expansions compared to the respective controls (Supplementary Figures S7F, G and S7K, L). These results did not support our hypothesis that SSB-induced repeat expansions were BIR-dependent, and at this point, we could not differentiate between DSB- and SSB-induced repeat expansions.

Taken together, our *in vitro* analyses revealed that there were at least two distinct modes of STR expansion; one mode was dependent on Msh2, a protein in the MMR pathway, and contributed to small continuous repeat expansion. Another mode was dependent on HDR, induced by artificially generated DSB/SSB, that partly contributed to large repeat expansion.

### *C9orf72* G_4_C_2_ repeat instability *in vivo*

**Figure 6.**
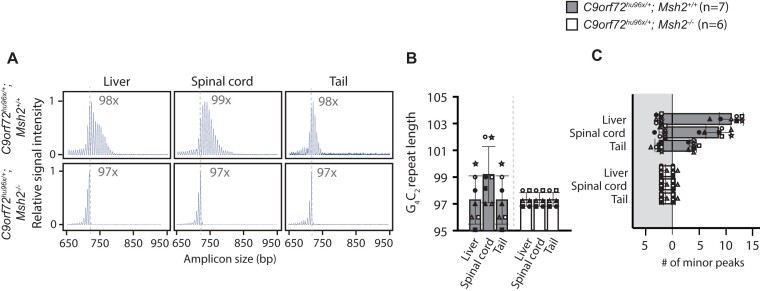
G_4_C_2_ somatic repeat instability in *C9orf72*^hu96x/+^*; Msh2*^−/^*^−^* mice. (**A**) Representative 3-primer RP-PCR CE traces from 2 months-old *C9orf72*^hu96x/+^*; Msh2*^+/+^ and *C9orf72*^hu96x/+^*; Msh2*^−/−^ tissues. The G_4_C_2_ repeat length (the G_4_C_2_ repeat copy number of the most abundant species corresponding to the highest peak in CE) is indicated in the graphs. Dotted lines represent the repeat length (97 copies of G_4_C_2_ repeats) in the *C9orf72^hu96x/+^; Msh2^−/−^* mES cells. (**B**) The G_4_C_2_ repeat length in *C9orf72*^hu96x/+^*; Msh2*^+/+^ and *C9orf72*^hu96x/+^*; Msh2*^−/−^ tissues. Each symbol represents individual sample. Vertical bars indicate mean values. (**C**) Quantification of the minor G_4_C_2_ repeat species. The number of peaks larger (white background), and smaller (grey background) than the highest peak shown. Same symbols in (B) and (C) (within the same group) represent samples derived from the same animal.

Next, we asked if these two modes of repeat expansion exist *in vivo*. To test the Msh2-dependent pathway, we analyzed selected tissues in two-month-old mice generated from *C9orf72*^hu96x/+^*; Msh2*^+/+^ or *C9orf72*^hu96x/+^*; Msh2*^−/−^ mES clones; liver as the most heterogenous tissue, spinal cord as the most disease-relevant tissue, and tail as one of the most stable tissues. Consistent with the CE traces from *C9orf72*^hu96x/+^*; Msh2*^−/−^ mES cells, the minor peaks beyond the highest peak were barely detectable in the *Msh2*^−/−^ background (Figure 6A and C). In addition, the increased heterogeneity in liver as well as overall change in repeat length in spinal cord in the *Msh2*^+/+^ background was not observed in *Msh2*^−/−^ background (Figure 6A–C). These data indicated that the *C9orf72* G_4_C_2_ somatic repeat expansion requires Msh2.

**Figure 7.**
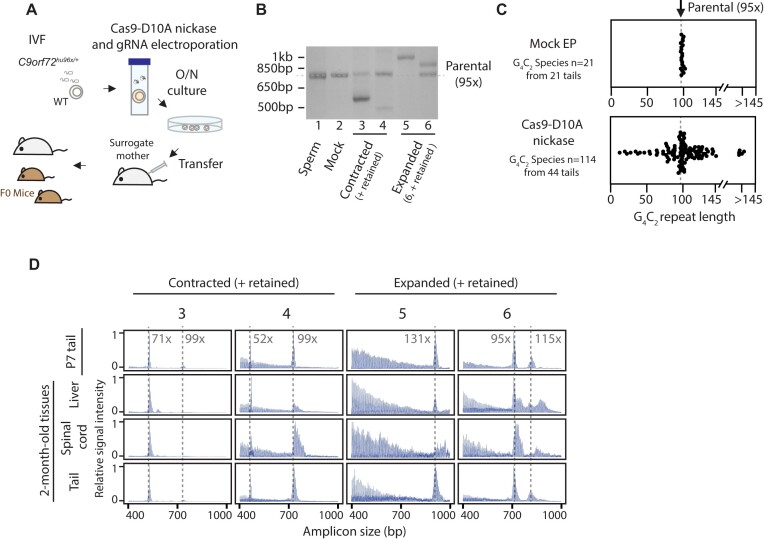
Repeat expansions by DNA SSB in one-cell embryos. (**A**) One-cell embryo Cas9-D10A nickase and gRNA injection workflow. *In vitro* fertilized embryos received Cas9-D10A nickase and C9-5′ gRNA by electroporation. Embryos were cultured overnight and transferred into pseudo-pregnant females. Tails and other tissues from resulting mice were analyzed at P7 (tails) and at 2 months of age (tissues). IVF, *in vitro* fertilization. Grey mice, surrogate mother. Brown mice, VelociMice (100% ES cell-derived F0 mice). (**B**) Gene-specific 2-primer PCR products following Cas9-D10A nickase and gRNA one-cell embryo injection. Gel electrophoresis of amplicons from representative P7 tail genomic DNA samples shown. Dotted line indicates the G_4_C_2_ repeat length in sperm donor (95 copies of G_4_C_2_). (**C**) The G_4_C_2_ repeat species in tails from mice generated by mock (top) or Cas9-D10A nickase (bottom) one-cell embryo injection. 114 G_4_C_2_ repeat species detected from total of 44 mice analyzed in nickase injection group. No mosaicism was observed in mock injection group. See Supplementary Figure S8 for detailed characterization in individual mouse. Dotted line indicates the G_4_C_2_ repeat length in sperm donor (95 copies of G_4_C_2_). (**D**) RP-PCR CE traces from *C9orf72^hu96x/+^* P7 tails and 2-month-old *C9orf72*^hu96x/+^ tissues that received Cas9-D10A and gRNA at one-cell embryo stage. The numbers on top of each column correspond to the lanes in (B). The dotted lines correspond to the peaks detected in P7 tail samples.

Since the deletion of *Rad51* leads to embryonic lethality in mice (86), we were unable to study *in vivo* HDR-dependent large repeat expansions as performed in cultured mES cells. Instead, we asked if a DNA DSB/SSB could induce large repeat expansions *in vivo*. When evaluating the CE traces from *C9orf72*^hu96x/+^; *Msh2^−/−^* tissues (Figure 6A), we were unable to detect any evidence of large-scale repeat expansions. We speculated that this was due to either a low frequency of spontaneous DSBs/SSBs or a high efficiency of DNA repair in somatic tissues. Therefore, to test if a DNA DSB/SSB also induces large repeat expansions *in vivo*, we introduced a 5′-SSB at the G_4_C_2_ repeats into one-cell *C9orf72*^hu96x/+^ embryos, transferred them into surrogate females, and analyzed the resulting mice (Figure 7A). We introduced a SSB instead of a DSB to avoid deleterious effects of DSBs *in vivo*. Analogous to what we observed in mES cells, we detected large repeat expansions and contractions in tails from *C9orf72*^hu96x/+^ mice derived from these targeted embryos (Figure 7B and C). Interestingly, mice exhibited a high degree of mosaicism, with up to five distinct G_4_C_2_ repeat species in a single tail (Supplementary Figure S8A). This mosaicism resulted in 114 distinct G_4_C_2_ repeat species in the 44 tails analyzed in the nickase-treated group, whereas no mosaicism was confirmed in mock-treated group (Supplementary Figure S8B). Consistent with the mES cell experiments, Cas9-D10A-nickase cleavage sites were seamlessly repaired (Supplementary Figure S8C). These changes, first interrogated at P7, persisted in multiple tissues at two months of age (examples shown in Figure 7D). Thus, a DNA SSB generated proximal to a repeat can induce G_4_C_2_ repeat expansions *in vivo*.

### Generation of larger G_4_C_2_ repeat alleles and intergenerational instability

**Figure 8.**
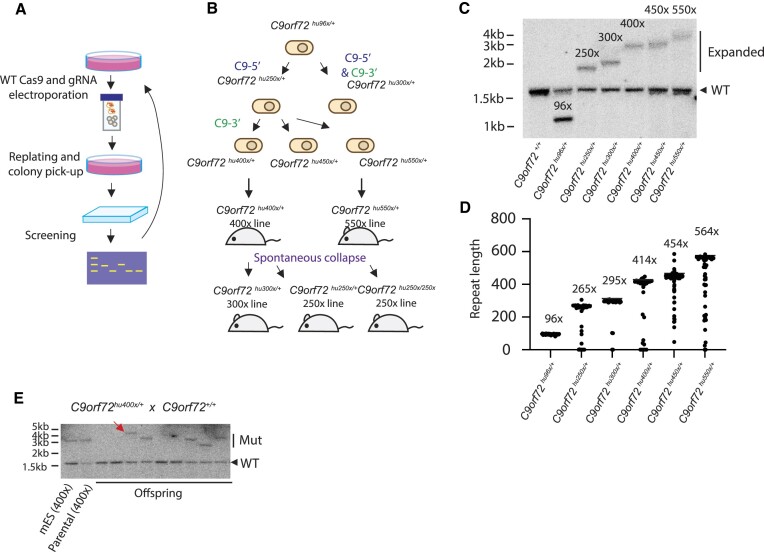
Generating larger *C9orf72* repeat alleles. (**A**,**B**) Workflow to generate larger *C9orf72* G_4_C_2_ repeat alleles in ES cells and mice. 96 copies of G_4_C_2_ repeats in *C9orf72*^hu96x/+^ mES cell clone was expanded by WT Cas9 and gRNAs. The repeat expansions were screened by 2-primer gene specific PCR, and selected clones were subjected to the second round of repeat expansion (A). *C9orf72*^hu250x/+^ and *C9orf72*^hu300x/+^ mES clones were obtained from *C9orf72*^hu96x/+^ mES cell clone, and *C9orf72*^hu400x/+^, *C9orf72*^hu450x/+^ and *C9orf72*^hu550x/+^ mES cell clones were obtained from *C9orf72*^hu250x/+^ mES cell clone. In mice, during colony maintenance, novel 250× and 300× lines were generated from 400× and 550× lines (B). (**C**) Southern blotting analysis of the repeat length of humanized *C9orf72* allelic series in mES cells. Estimated G_4_C_2_ repeat length indicated. Note the Southern blotting probe was designed to recognize both WT and humanized alleles. (**D**) STRique analysis of the humanized *C9orf72* allelic series in mES cells using nanopore sequencing reads. Most frequently called repeat length shown as horizontal bars and numbers. Number of nanopore sequence reads used for the STRique analysis were, *C9orf72*^hu96x/+^ (*n* = 79), *C9orf72*^hu250x/+^ (*n* = 50), *C9orf72*^hu300x/+^ (*n* = 44), *C9orf72*^hu400x/+^ (*n* = 33), *C9orf72*^hu450x/+^ (*n* = 143) and *C9orf72*^hu550x/+^ (*n* = 50). (**E**) A representative *C9orf72* repeat length analysis using tail biopsies by Southern blotting. A litter of offspring from breeding pair *C9orf72*^hu400x/+^ male and WT female mice were analyzed. One of the offspring had approximately 700 copies of G_4_C_2_ repeats (red arrow).

Though quite rare, large intergenerational repeat expansions in mouse RED models have been noted, including the *C9orf72* G_4_C_2_ repeats (19,70,87,88). These are sporadic events, and it is not clear what factors cause large intergenerational repeat expansions. Expanded STRs accumulate DNA DSBs in a length-dependent manner both in yeast and mammalian genomes (89,90). If this holds true for the *C9orf72* G_4_C_2_ repeats, then we could recapitulate dynamic intergenerational repeat instability using larger *C9orf72* G_4_C_2_ repeat alleles. Because we were unable to find spontaneously repeat-expanded alleles by breeding *C9orf72*^hu96x/+^ mice (Figure 2D), we induced large-scale expansions via DNA DSBs (Figure 3) and searched for mES cell clones that contain even larger repeat alleles with which to further characterize *C9orf72* G_4_C_2_ intergenerational repeat expansions (Figure 8A). We explored the consequence of single 5′-DSB and dual DSBs (both 5′ and 3′) on the *C9orf72*^hu96x/+^ mES cells (schematized in Figure 8B). Screening of ES cells that received 5′-DSB by Cas9 and C9-5′ identified a clone with approximately 250 copies of the G_4_C_2_ repeats (*C9orf72*^hu250x/+^). From the dual DSB experiment, we obtained a clone that had even larger copy numbers of the repeat, approximately 300 copies of G_4_C_2_ (*C9orf72*^hu300x/+^), but this clone showed one base pair deletion at the 3′-DSB (Supplementary Figure S9B). Since the 5′ Cas9 cleavage site was intact in the newly identified *C9orf72*^hu250x/+^ clone, we expanded the G_4_C_2_ repeats in this clone further by introducing a 3′-DSB at the repeats (Figure 8B). We found three additional clones from the second round of DSB introduction with approximately 400, 450 and 550 copies of G_4_C_2_ (*C9orf72*^hu400x/+^, *C9orf72*^hu450x/+^, and *C9orf72*^hu550x/+^) as confirmed by Southern blotting (Figure 8C). The repeat length estimation by STRique analysis using nanopore sequencing reads was similar to the estimation by Southern blotting (Figure 8D). 3′-DSB-induced expansion frequency from 250 copies of G_4_C_2_ (13.3%) was higher than that from 96 copies (3.4%) or 31 copies (0%), further supporting the notion that expansion frequency and starting repeat length are positively correlated at this locus (Table 1). We then generated and bred mice derived from *C9orf72*^hu400x/+^ and *C9orf72*^hu550x/+^ clones (400× line and 550× line, respectively, Figure 8B) to test intergenerational repeat instability. Unlike the case of *C9orf72*^hu96x/+^ mice, both of the newly generated lines were less stable and thereby produced progeny harbouring alleles with fewer copies of the G_4_C_2_ repeats. We subsequently examined the intergenerational stability of the entire allelic series (96×, 250×, 300×, 400× and 550× lines) by analyzing the G_4_C_2_ repeat length in the tail biopsies from the neonates. Using Southern blotting analysis, we categorized rearrangements conservatively into three types: (A) contraction; the G_4_C_2_ repeat length is 0.5-fold or less than the parental one; (B) expansion: 1.5-fold or more than the parental one and (C) retention: between 0.5- and 1.5-fold of the parental one. The G_4_C_2_ repeats in mice up to 300 copies were relatively stable, with only minor intergenerational changes (Table 2). However, in mice with >400 copies of the G_4_C_2_ repeats at the humanized *C9orf72* locus, the repeats became less stable, with 3.4–6.3% of offspring showing contracted repeat lengths, regardless of the parental sex (Table 2). Notably, one mouse showed a clear expansion from the parental 400 copies to approximately 700 copies (Figure 8E). Thus, we demonstrated in mice that larger G_4_C_2_ repeats exhibited greater intergenerational repeat instability, trending toward contraction, even without exogenously introducing DNA DSB or SSB.

**Table 2.** Intergenerational repeat instability in humanized *C9orf72* mice

Parental repeat length	Parental sex	# of offspring mice analyzed	Offspring repeat length	Retention (>0.5-fold, <1.5-fold)	Contraction (<0.5-fold)	Expansion (>1.5-fold)
95×	Male (Sperm)	21	94×–100×	21 (100%)	0 (0%)	0 (0%)
95×–97×	Male	72	93×–102×	72 (100%)	0 (0%)	0 (0%)
95×–97×	Female	62	92×–100×	62 (100%)	0 (0%)	0 (0%)
95× + Cas9–D10A	Male (Sperm)	44	11×-150×	42 (95.5%)*	5 (11.4%)*	6 (13.6%)*
250×	Male	36	200×–300×	36 (100%)	0 (0%)	0 (0%)
250×	Female	41	200×–300×	41 (100%)	0 (0%)	0 (0%)
300×	Female	16	250×–350×	16 (100%)	0 (0%)	0 (0%)
400×	Male	91	100×–700×	85 (93.4%)	5 (5.5%)	1 (1.1%)
400×	Female	48	100×–450×	45 (93.7%)	3 (6.3%)	0 (0%)
550×	Male	87	100×–650×	84 (96.5%)	3 (3.4%)	0 (0%)
550×	Female	71	100×–650×	68 (92.9%)	3 (4.2%)	0 (0%)

*The number (percentage) of mice with indicated type of instability. Sum of these numbers (percentages) exceeded total number of mice analyzed due to the mosaicism.

## Discussion

Disease-causing STRs exhibit somatic instability as well as intergenerational instability. Multiple studies have shown the significant contribution of proteins in MMR pathway to somatic instability, while the molecular mechanism of intergenerational instability, especially those that accompany major changes in STR length, is largely unknown. In this study, we generated novel humanized *C9orf72* alleles, a model for familial ALS/FTD associated with the G_4_C_2_ repeat expansion, and studied both types of repeat instability. We found that a DNA DSB or SSB could serve as a trigger for large-scale repeat expansion, which provided us an important molecular clue for the understanding of STR instability.

We used gene targeting to create our humanized *C9orf72* alleles, by inserting human *C9orf72* intron 1 and expanded G_4_C_2_ repeats in the mouse ortholog. Unlike other BAC transgene-based humanized *C9orf72* models (49–52), inserting the expanded repeats into the native locus should remove any positional effects from random integrations or overexpression due to multi-copy inserts. Because of these advantages, our model may better recapitulate the behaviour of human G_4_C_2_ repeats, and possibly ALS/FTD-like disease phenotypes as well.

Our analysis of the G_4_C_2_ repeats in mES cells and tissues in unperturbed conditions revealed that these repeats exhibited minor but continuous small-scale expansions that accumulated in the genome. These expansions were highly variable among tissues and dependent on Msh2, a protein in the MMR pathway shown to be involved in the somatic repeat expansions in other RED mouse models (Figures 1C–D, 2A–C, 5A–D and 6A–C) (23,25,26,32). Disease-causing STRs in published RED mouse models, including CTG repeats in DM1, CGG repeats in FXS, and CAG repeats in HD, consistently expanded during the mouse lifetime in a tissue-dependent manner, and these expansions were associated with a subset of proteins in the MMR pathway (21,23,25–27,32,91,92). Hence, it is possible that not only Msh2, but also a subset of proteins in the MMR pathway, contribute to somatic repeat expansions of *C9orf72* G_4_C_2_ repeats as well. In our mouse model, the G_4_C_2_ repeats expanded up to approximately 125% of estimated inherited repeat length in liver, the least stable tissue, by the age of 6 months (Figure 2A–C). Interestingly, the same magnitude of expansions (10–40% increase) was observed in the livers at 6–12 month of age among other RED models when approximately 100 copies of repeats were inserted (32,91,92). If these STRs in the human genome expand at a similar speed as in mouse cells, then, because of human's longer life span, these continuous small expansions may still give rise to pathogenic larger repeat alleles. Human genetic studies revealed the association of somatic repeat expansions and disease onset in HD and DM1 (36,37). These data suggest that somatic repeat expansions might be a common contributor to RED pathogenesis in humans.

In some ALS/FTD patients, the *C9orf72* repeats were robustly expanded in CNS but not in peripheral tissues, which raised the possibility that somatic repeat expansions also play a critical role in ALS/FTD pathogenesis (45–47). However, the CNS-specific repeat expansion is not a common feature among patients, which makes it difficult to assess the contribution of somatic repeat expansion. As genotype-phenotype relationship is further characterized, our novel ALS/FTD mouse model will become a useful tool to molecularly dissect the role of the somatic expansions on disease pathogenesis (manuscript in preparation).

To date, because of the technical hurdles measuring accurate repeat length, reliable prediction of the repeat length in CNS, by collecting and analyzing samples from typical collection sites such as blood or saliva has not been established (45–47,93–97). In our mouse models, the degree of instability was different between whole brain/spinal cord and peripheral tissues (Figure 2A–C), which confirmed the need for tissue-specific characterization of the somatic repeat instability. Systematic characterization of the G_4_C_2_ repeat somatic instability in our humanized *C9orf72* allelic series, across tissues and in longitudinal analyses using various starting repeat lengths, will enable deeper understanding of the behavior of the G_4_C_2_ repeats. Also, a comprehensive study which captures repeat instability and its corresponding molecular phenotypes in mice should be informative for translational research.

The major repeat expansions induced by DNA DSB/SSB and their application for disease modelling (Figures 3 and 8) represents a new paradigm to create RED models in a genetically-tractable mammalian system. The magnitude of repeat expansion seen in humans is difficult to reproduce in the mouse models with some exceptions (19,70,87,88). Hence, it has been difficult to generate RED mouse models containing large STRs. In our effort to identify factors that could trigger a major change in repeat length in the mammalian genome, we found that a DNA DSB and a SSB, introduced by WT Cas9 and Cas9-D10A nickase respectively, proximal to the disease-causing STRs was able to induce a significant (more than 1.5-fold) magnitude of repeat expansions (Figure 3). Using this procedure, we can now introduce large repeat alleles in the mammalian genome that were difficult to achieve by conventional targeting vector-based approaches. The allelic series generated in this manner (Figure 8) might be one of the best sample sets with which to study the impact of the STR length in an *in vivo* disease model, because the only genetic difference among resulting mice bred from these clones is the repeat length. We cannot rule out the possibility of mutations generated by Cas9 off-targeting effects, but these mutations can be segregated by breeding. We are currently analyzing phenotypes of the mouse models generated in this study to better characterize genotype-phenotype relationships of REDs including ALS/FTD associated with expansion of *C9orf72* G_4_C_2_, *TCF4* CTG and *FXN* GAA repeats.

One longstanding question is if a mechanism exists that can achieve major repeat expansions as a single event, distinct from the mechanism of minor repeat expansions that add only small increase in repeat length per event (2,4). In our study, introduction of a DSB or a SSB resulted in a major change in *C9orf72* G_4_C_2_ repeat length, up to 2.5-fold, in a Msh2-independent, and Rad51-dependent manner, presumably via HDR pathway (Figures 3, 5, 8, and Table 1). We also observed that Msh2-dependent *C9orf72* G_4_C_2_ repeat expansion, which continuously induced minor changes in repeat length that accumulated in the genome, was the major driver of somatic repeat expansion in mouse tissues in unperturbed condition (Figures 2 and 6). As discussed above, the common contribution of a subset of MMR pathway proteins to somatic repeat expansions observed in multiple RED models suggests that this may also be the case for *C9orf72* G_4_C_2_ repeats. Collectively, we speculate that there are two distinct modes of *C9orf72* G_4_C_2_ repeat expansion; one is DSB/SSB-induced HDR-dependent expansion that could cause major changes in repeat length per expansion event, and the other is an expansion dependent on a subset of proteins in the MMR pathway that exert minor changes in repeat length per expansion event. Using a yeast model, Kim *et al.* showed that the large CAG repeat expansions occurred via BIR, a Rad51-dependent error-prone HDR pathway that repairs one-ended DSB (39). These experimental systems, the engineered yeast genome, or exogenous Cas9 delivery into the engineered mouse genome by us, may not be relevant to pathophysiological conditions, and therefore, whether the large repeat expansions occur similarly in the human genome requires further investigations. Nevertheless, these observations point to the existence of the large-scale repeat expansions achieved via HDR-dependent pathway, which is distinct from continuous small repeat expansions dependent on proteins in MMR pathway. Biological relevance is an important aspect of our future studies.

It is tempting to speculate that DSB/SSB contribute to large-scale repeat expansions as well as contractions seen in human pedigrees. We observed length-dependent intergenerational repeat instability (Table 2): the G_4_C_2_ repeats above 400 copies trended toward contraction; while, through extensive breeding, we confirmed a case of large-scale expansion from 400 copies in the male parent to 700 copies in an offspring (Figure 8E). Recently, two additional cases of large-scale expansions were found during colony maintenance. STRs are known as ‘difficult to replicate’ regions (98,99). Non-B structure DNA, such as hairpin or G-quadruplex, formed at the repetitive sequence can stall the DNA replication fork which, if not resolved immediately, can lead to a DNA fork collapse and thereby generate DNA DSBs (100,101). Indeed, studies indicate that large STRs are the sites of DSB *in vivo* in yeast as well as mammalian models (89,90). In addition, reactive oxygen species (ROS), a cellular stress marker, or γ-H2AX, a DNA damage marker, were elevated in *C9orf72* repeat-expanded ALS patient tissues as well as in patient-derived iPS cells when cultured for a prolonged time (102,103). The intergenerational repeat instability we observed in our mouse model is consistent with the hypothesis; DSB/SSB occurred in larger repeats more frequently compared with shorter repeats, which triggered repeat length dependent instability. However, we do not have direct evidence of spontaneous DSB/SSB-induced repeat expansions via HDR pathway in either cultured cells or in mice, representing an important hypothesis to test in future work.

Another unanswered question is why the frequency or the magnitude of repeat expansions, during intergenerational transmission, is different between human and mouse. For example, >80 copies of CTG repeats exhibit strong bias toward further expansions through maternal transmission that could result in a major increase in repeat length. DM1 mouse models do not show the same magnitude of expansions even when they have >300 copies of repeats in female animals, even though high frequency of repeat expansions were observed (14–16,19). If DSB/SSB-induced repeat expansion contributes to the repeat expansions in human pedigree, does it explain the difference in these two species? As shown in Table 1, the frequency of repeat expansions after DSB was positively correlated with the starting G_4_C_2_ repeat length in mES cells. Since HDR was a key driver for the large-scale repeat expansions (Figure 5F–H), the difference in repairing large repeats between human and mouse such as choice of DNA repair pathways, may explain the difference in intergenerational repeat instability. We are currently generating mouse and human cell lines with similar G_4_C_2_ repeat lengths to compare DNA repair mechanisms between species after DSB/SSB.

**Figure 9.**
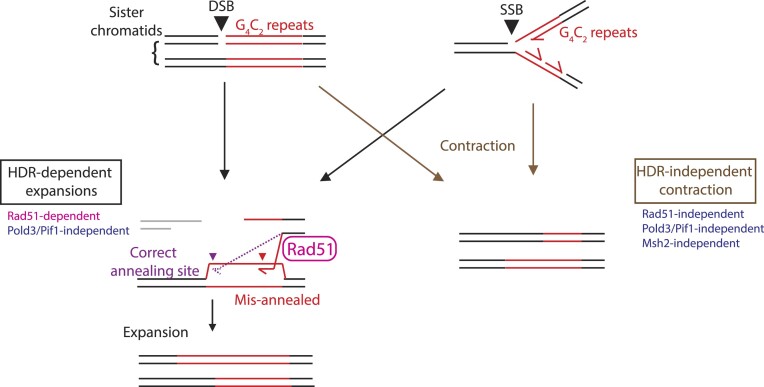
Model of the repeat expansions and contractions by a DNA DSB or SSB. Following a DSB introduction adjacent to the G_4_C_2_ repeats (repeats shown in red), a broken DNA end with G_4_C_2_ repeat sequence, after end-processing, invades into the sister-chromatid but can misalign within the G_4_C_2_ repeats at ‘out-of-register’ position, which could result in the repeat expansion. A SSB during S phase can generate one-ended or two-ended DSB (one of the ends shown in light grey lines indicating possibilities of both one-ended and two-ended DSB). Misalignment during HDR could also explain SSB-induced expansions. These SSB-induced expansions, as well as DSB-induced expansions, occurred in a Pold3- or Pif1-independent manner. DSB and SSB also induced repeat contractions, but they occurred mainly via non-HDR pathways.

Inhibition of *Rad51* significantly decreased the frequency of DSB-induced *C9orf72* G_4_C_2_ repeat expansions (Figure 5F–H), suggesting these expansions were HDR-dependent. When a DSB with two broken ends is repaired by the HDR pathway, it is preferentially achieved by error-free HDR pathways, SDSA or DSBR (78,104). In these pathways, broken DNA ends are resected, coated by Rad51, and ssDNA-Rad51 nucleofilament searches for and invades a donor template, typically the sister chromatid, forming a D-loop where strand synthesis initiates (78,105). When a DSB is introduced adjacent to *C9orf72* G_4_C_2_ repeats and repaired by HDR, the processed DNA-end with G_4_C_2_ repetitive sequence, after strand invasion, may mis-align ‘out-of-register’ to an incorrect position within the stretch of G_4_C_2_ repeats in the sister chromatid. Initiation of strand synthesis from the mis-aligned position may then cause repeat expansion (Figure 9). A SSB during S phase, if it encounters replication fork, can generate either one-ended or two-ended DSB depending on the locations of replication origins (79,106) (Figure 9). Since inhibition of *Rad51* also significantly reduced the frequency of SSB-induced repeat expansions, the same mis-alignment within the G_4_C_2_ repeats discussed above can also explain SSB-induced expansions. An error-prone HDR pathway BIR can repair one-ended DSB induced by SSB, as well as two-ended DSB when coordination of two broken ends fails (78,104). Suppression of *Pold3* by siRNA or knocking out *Pif1*, both of which are reportedly involved in BIR (40,41,83–85), did not alter the frequency of DSB-induced nor SSB-induced repeat expansions (Supplementary Figure S7C–L), suggesting these repeat expansions were BIR-independent events. However, mammalian BIR has not been fully characterized and hence alternative pathways might exist. Since it is difficult to knock down *Rad51* completely during the long process of DNA DSB repair by recombination (107), it is reasonable to observe partial inhibition of the repeat expansions (Figure 5F–H). However, at this point, we cannot exclude the involvement of other Rad51-independent mechanisms, such as microhomology-mediated BIR (MMBIR), Rad51-independent mitotic DNA synthesis, or strand slippage (6,38,84), to explain the large repeat expansions resulting from a DSB/SSB. In contrast to repeat expansions, repeat contractions after a DSB/SSB should be achieved mainly by non-HDR pathways as Rad51 knock-down did not alter the frequency of repeat contractions (Figures 5F–H and 9).

At *C9orf72* G_4_C_2_ repeats, in both mES cells and in one-cell embryos, Cas9-D10A nickase did not produce indels at the cleavage site even when repeats were expanded or contracted (examples shown in Supplementary Figures S4A and S8C). In these rearranged clones, because gRNA recognition sequences were intact, the nickase could act on DNA repeatedly, and that might have produced a high degree of mosaicism during cell divisions in culture or embryogenesis. Somatic repeat length mosaicism is commonly seen in ALS/FTD patients (44). The contribution of naturally occurring SSBs, during early development, to mosaicism presents an interesting subject for our future studies.

At *C9orf72* G_4_C_2_ repeats in our model, frequency of expansions and contractions following DSB/SSB introduction varied depending on the starting repeat length as well as location of the DNA lesions (Figure 3 and Table 1). At *Tcf4* CTG or *Fxn* GAA repeats, 5′-DSB or 3′-DSB respectively induced repeat expansions, but corresponding SSB did not (Figure 4 and Table 1). The frequency of repeat contractions after DSB or SSB was also different among these repeats. Since G_4_C_2_, CTG, and GAA repeats are predicted to have different secondary structures (G-quadruplex, hairpins, or H-DNA respectively (2,108,109)), these secondary structures might impose different challenges on DNA repair after strand breakage, and therefore, might lead to locus-dependent outcome. Because of the variations in frequency or pattern of repeat length alterations, at this point, we do not have a general model that can predict the consequence of the genome rearrangements after DSB/SSB across disease causing STRs. Further investigation is required for the comprehensive understanding of the behavior of expanded STRs.

Some REDs including DM1 or HD, where repeat length is strongly correlated with disease onset/severity, exhibit genetic anticipation, a biological phenomenon in which disease onset becomes earlier, and disease severity becomes stronger, than in the previous generation (2,3). To date, it is not yet clear whether genetic anticipation also plays a role in the *C9orf72* ALS/FTD, partly due to difficulties characterizing this STR (44,47). However, our current understanding from human genetic studies suggests that larger *C9orf72* G_4_C_2_ repeats tend to contract rather than expand between generations (44,95,97). Consistent with this notion, our experiments also demonstrate that alleles which contain more than 400 copies of *C9orf72* G_4_C_2_ repeats tend to contract more frequently than they expand (Table 2). Though we need further investigation regarding the species differences between human and mice, our humanized RED models including *C9orf72* G_4_C_2_, *Tcf4* CTG and *Fxn* GAA models, may help us obtaining further insights into the variation of genetic anticipation among REDs.

With emerging techniques to engineer genomic DNA using programable RNAs and endonucleases, multiple novel approaches were proposed to treat human diseases including REDs. One of these proposals is excision of the expanded intronic STRs by introducing DSBs at both sides of the STRs (110,111). Studies showed that introducing DSBs by WT Cas9 caused large deletion at any given locus, although this was not major outcome (112,113). In our hands, when DSB was introduced in repeat expanded humanized *C9orf72* mES cells, large deletions were observed consistently (Figure 3 and Table 1). These observations suggested that the Crispr-based excision approach might remove not only intronic STRs but also additional sequences around the STR, and potentially altering the sequence near the surrounding exons. Also, DSBs induced by Cas9 could expand the intronic STRs as we observed in this study (Figures 3 and 4, and Table 1), which could potentially further worsen disease severity. Since DSB-induced repeat expansions were dependent on HDR (Figure 5F–H) that is not highly active in post-mitotic cells such as mature neurons, the most vulnerable cell type of many REDs, it is less likely that DSBs induce repeat expansions in this cell type. Nevertheless, a careful evaluation may be required for this type of therapeutic application.

In summary, we demonstrated the *C9orf72* G_4_C_2_ repeats exhibited repeat instability both somatically and intergenerationally using a novel mouse ALS model. We presented evidence that DNA DSB/SSB have a profound impact on repeat instability. These findings provide molecular clues to better understand the dynamic behaviour of STRs in the mammalian genome.

## Supplementary Material

gkae250_Supplemental_File

## Data Availability

The data underlying this article are available in the article and in its online supplementary material.

## Accession numbers

This study includes no data deposited in external repositories.

## Notes

Present address: Daisuke Kajimura, Regeneron Pharmaceuticals, Tarrytown, NY 10591, USA.

## References

[1] Lander E.S., Linton L.M., Birren B., Nusbaum C., Zody M.C., Baldwin J., Devon K., Dewar K., Doyle M., FitzHugh W. et al. Initial sequencing and analysis of the human genome. Nature. 2001; 409:860–921. 11237011 10.1038/35057062

[2] Khristich A.N., Mirkin S.M. On the wrong DNA track: molecular mechanisms of repeat-mediated genome instability. J. Biol. Chem. 2020; 295:4134–4170. 32060097 10.1074/jbc.REV119.007678 PMC7105313

[3] Depienne C., Mandel J.L. 30 years of repeat expansion disorders: what have we learned and what are the remaining challenges?. Am. J. Hum. Genet. 2021; 108:764–785. 33811808 10.1016/j.ajhg.2021.03.011 PMC8205997

[4] McMurray C.T. Mechanisms of trinucleotide repeat instability during human development. Nat. Rev. Genet. 2010; 11:786–799. 20953213 10.1038/nrg2828 PMC3175376

[5] Dion V. Tissue specificity in DNA repair: lessons from trinucleotide repeat instability. Trends Genet. 2014; 30:220–229. 24842550 10.1016/j.tig.2014.04.005

[6] Usdin K., House N.C., Freudenreich C.H. Repeat instability during DNA repair: Insights from model systems. Crit. Rev. Biochem. Mol. Biol. 2015; 50:142–167. 25608779 10.3109/10409238.2014.999192 PMC4454471

[7] Heitz D., Devys D., Imbert G., Kretz C., Mandel J.L. Inheritance of the fragile X syndrome: size of the fragile X premutation is a major determinant of the transition to full mutation. J. Med. Genet. 1992; 29:794–801. 1453430 10.1136/jmg.29.11.794 PMC1016175

[8] Hagerman R.J., Hagerman P.J. The fragile X premutation: into the phenotypic fold. Curr. Opin. Genet. Dev. 2002; 12:278–283. 12076670 10.1016/s0959-437x(02)00299-x

[9] Lozano R., Rosero C.A., Hagerman R.J. Fragile X spectrum disorders. Intractable Rare Dis. Res. 2014; 3:134–146. 25606363 10.5582/irdr.2014.01022 PMC4298643

[10] Wheeler A.C., Bailey D.B. Jr, Berry-Kravis E., Greenberg J., Losh M., Mailick M., Mila M., Olichney J.M., Rodriguez-Revenga L., Sherman S et al. Associated features in females with an FMR1 premutation. J. Neurodev. Disord. 2014; 6:30. 25097672 10.1186/1866-1955-6-30 PMC4121434

[11] Hayward B.E., Usdin K. Mechanisms of genome instability in the fragile X-related disorders. Genes (Basel). 2021; 12:1633. 34681027 10.3390/genes12101633 PMC8536109

[12] Brook J.D., McCurrach M.E., Harley H.G., Buckler A.J., Church D., Aburatani H., Hunter K., Stanton V.P., Thirion J.P., Hudson T. et al. Molecular basis of myotonic dystrophy: expansion of a trinucleotide (CTG) repeat at the 3' end of a transcript encoding a protein kinase family member. Cell. 1992; 68:799–808. 1310900 10.1016/0092-8674(92)90154-5

[13] Tome S., Gourdon G. DM1 phenotype variability and triplet repeat instability: challenges in the development of new therapies. Int. J. Mol. Sci. 2020; 21:457. 31936870 10.3390/ijms21020457 PMC7014087

[14] Morales F., Vasquez M., Cuenca P., Campos D., Santamaria C., Del Valle G., Brian R., Sittenfeld M., Monckton D.G. Parental age effects, but no evidence for an intrauterine effect in the transmission of myotonic dystrophy type 1. Eur. J. Hum. Genet. 2015; 23:646–653. 25052313 10.1038/ejhg.2014.138 PMC4402617

[15] Joosten I.B.T., Hellebrekers D., de Greef B.T.A., Smeets H.J.M., de Die-Smulders C.E.M., Faber C.G., Gerrits M.M. Parental repeat length instability in myotonic dystrophy type 1 pre- and protomutations. Eur. J. Hum. Genet. 2020; 28:956–962. 32203199 10.1038/s41431-020-0601-4 PMC7316980

[16] Han J.Y., Jang W., Park J. Intergenerational influence of gender and the DM1 phenotype of the transmitting parent in Korean myotonic dystrophy type 1. Genes (Basel). 2022; 13:1465. 36011377 10.3390/genes13081465 PMC9408469

[17] Mankodi A., Logigian E., Callahan L., McClain C., White R., Henderson D., Krym M., Thornton C.A. Myotonic dystrophy in transgenic mice expressing an expanded CUG repeat. Science. 2000; 289:1769–1773. 10976074 10.1126/science.289.5485.1769

[18] Seznec H., Lia-Baldini A.S., Duros C., Fouquet C., Lacroix C., Hofmann-Radvanyi H., Junien C., Gourdon G. Transgenic mice carrying large human genomic sequences with expanded CTG repeat mimic closely the DM CTG repeat intergenerational and somatic instability. Hum. Mol. Genet. 2000; 9:1185–1194. 10767343 10.1093/hmg/9.8.1185

[19] Gomes-Pereira M., Foiry L., Nicole A., Huguet A., Junien C., Munnich A., Gourdon G. CTG trinucleotide repeat “big jumps”: large expansions, small mice. PLoS Genet. 2007; 3:e52. 17411343 10.1371/journal.pgen.0030052 PMC1847694

[20] Schmidt M.H.M., Pearson C.E. Disease-associated repeat instability and mismatch repair. DNA Repair (Amst.). 2016; 38:117–126. 26774442 10.1016/j.dnarep.2015.11.008

[21] Wheeler V.C., Dion V. Modifiers of CAG/CTG repeat instability: insights from mammalian models. J. Huntingtons Dis. 2021; 10:123–148. 33579861 10.3233/JHD-200426 PMC7990408

[22] Zhao X., Kumari D., Miller C.J., Kim G.Y., Hayward B., Vitalo A.G., Pinto R.M., Usdin K. Modifiers of somatic repeat instability in mouse models of Friedreich ataxia and the fragile X-related disorders: implications for the mechanism of somatic expansion in Huntington's disease. J Huntingtons Dis. 2021; 10:149–163. 33579860 10.3233/JHD-200423 PMC7990428

[23] Manley K., Shirley T.L., Flaherty L., Messer A. Msh2 deficiency prevents in vivo somatic instability of the CAG repeat in Huntington disease transgenic mice. Nat. Genet. 1999; 23:471–473. 10581038 10.1038/70598

[24] van den Broek W.J., Nelen M.R., Wansink D.G., Coerwinkel M.M., te Riele H., Groenen P.J., Wieringa B. Somatic expansion behaviour of the (CTG)n repeat in myotonic dystrophy knock-in mice is differentially affected by Msh3 and Msh6 mismatch-repair proteins. Hum. Mol. Genet. 2002; 11:191–198. 11809728 10.1093/hmg/11.2.191

[25] Savouret C., Brisson E., Essers J., Kanaar R., Pastink A., te Riele H., Junien C., Gourdon G. CTG repeat instability and size variation timing in DNA repair-deficient mice. EMBO J. 2003; 22:2264–2273. 12727892 10.1093/emboj/cdg202 PMC156074

[26] Wheeler V.C., Lebel L.A., Vrbanac V., Teed A., te Riele H., MacDonald M.E. Mismatch repair gene Msh2 modifies the timing of early disease in Hdh(Q111) striatum. Hum. Mol. Genet. 2003; 12:273–281. 12554681 10.1093/hmg/ddg056

[27] Dragileva E., Hendricks A., Teed A., Gillis T., Lopez E.T., Friedberg E.C., Kucherlapati R., Edelmann W., Lunetta K.L., MacDonald M.E. et al. Intergenerational and striatal CAG repeat instability in Huntington's disease knock-in mice involve different DNA repair genes. Neurobiol. Dis. 2009; 33:37–47. 18930147 10.1016/j.nbd.2008.09.014 PMC2811282

[28] Bourn R.L., De Biase I., Pinto R.M., Sandi C., Al-Mahdawi S., Pook M.A., Bidichandani S.I. Pms2 suppresses large expansions of the (GAA.TTC)n sequence in neuronal tissues. PLoS One. 2012; 7:e47085. 23071719 10.1371/journal.pone.0047085 PMC3469490

[29] Pinto R.M., Dragileva E., Kirby A., Lloret A., Lopez E., St Claire J., Panigrahi G.B., Hou C., Holloway K., Gillis T. et al. Mismatch repair genes Mlh1 and Mlh3 modify CAG instability in Huntington's disease mice: genome-wide and candidate approaches. PLoS Genet. 2013; 9:e1003930. 24204323 10.1371/journal.pgen.1003930 PMC3814320

[30] Tome S., Manley K., Simard J.P., Clark G.W., Slean M.M., Swami M., Shelbourne P.F., Tillier E.R., Monckton D.G., Messer A. et al. MSH3 polymorphisms and protein levels affect CAG repeat instability in Huntington's disease mice. PLoS Genet. 2013; 9:e1003280. 23468640 10.1371/journal.pgen.1003280 PMC3585117

[31] Ezzatizadeh V., Sandi C., Sandi M., Anjomani-Virmouni S., Al-Mahdawi S., Pook M.A. MutLalpha heterodimers modify the molecular phenotype of Friedreich ataxia. PLoS One. 2014; 9:e100523. 24971578 10.1371/journal.pone.0100523 PMC4074104

[32] Lokanga R.A., Zhao X.N., Usdin K. The mismatch repair protein MSH2 is rate limiting for repeat expansion in a fragile X premutation mouse model. Hum. Mutat. 2014; 35:129–136. 24130133 10.1002/humu.22464 PMC3951054

[33] Zhao X.N., Kumari D., Gupta S., Wu D., Evanitsky M., Yang W., Usdin K. Mutsbeta generates both expansions and contractions in a mouse model of the fragile X-associated disorders. Hum. Mol. Genet. 2015; 24:7087–7096. 26420841 10.1093/hmg/ddv408 PMC4654059

[34] Zhao X.N., Lokanga R., Allette K., Gazy I., Wu D., Usdin K. A MutSbeta-dependent contribution of MutSalpha to repeat expansions in fragile X premutation mice?. PLoS Genet. 2016; 12:e1006190. 27427765 10.1371/journal.pgen.1006190 PMC4948851

[35] Genetic Modifiers of Huntington's Disease, C. Identification of genetic factors that modify clinical onset of Huntington's disease. Cell. 2015; 162:516–526. 26232222 10.1016/j.cell.2015.07.003 PMC4524551

[36] Genetic Modifiers of Huntington’s Disease (GeM-HD) Consortium CAG repeat not polyglutamine length determines timing of Huntington's disease onset. Cell. 2019; 178:887–900. 31398342 10.1016/j.cell.2019.06.036 PMC6700281

[37] Flower M., Lomeikaite V., Ciosi M., Cumming S., Morales F., Lo K., Hensman Moss D., Jones L., Holmans P., Investigators T.-H. et al. MSH3 modifies somatic instability and disease severity in Huntington's and myotonic dystrophy type 1. Brain. 2019; 142:1876–1886. 31216018 10.1093/brain/awz115 PMC6598626

[38] Carvalho C.M., Lupski J.R. Mechanisms underlying structural variant formation in genomic disorders. Nat. Rev. Genet. 2016; 17:224–238. 26924765 10.1038/nrg.2015.25 PMC4827625

[39] Kim J.C., Harris S.T., Dinter T., Shah K.A., Mirkin S.M. The role of break-induced replication in large-scale expansions of (CAG)(n)/(CTG)(n) repeats. Nat. Struct. Mol. Biol. 2017; 24:55–60. 27918542 10.1038/nsmb.3334 PMC5215974

[40] Kononenko A.V., Ebersole T., Vasquez K.M., Mirkin S.M. Mechanisms of genetic instability caused by (CGG)(n) repeats in an experimental mammalian system. Nat. Struct. Mol. Biol. 2018; 25:669–676. 30061600 10.1038/s41594-018-0094-9 PMC6082162

[41] Garribba L., Bjerregaard V.A., Goncalves Dinis M.M., Ozer O., Wu W., Sakellariou D., Pena-Diaz J., Hickson I.D., Liu Y. Folate stress induces SLX1- and RAD51-dependent mitotic DNA synthesis at the fragile X locus in human cells. Proc. Natl. Acad. Sci. U.S.A. 2020; 117:16527–16536. 32601218 10.1073/pnas.1921219117 PMC7368274

[42] DeJesus-Hernandez M., Mackenzie I.R., Boeve B.F., Boxer A.L., Baker M., Rutherford N.J., Nicholson A.M., Finch N.A., Flynn H., Adamson J. et al. Expanded GGGGCC hexanucleotide repeat in noncoding region of C9ORF72 causes chromosome 9p-linked FTD and ALS. Neuron. 2011; 72:245–256. 21944778 10.1016/j.neuron.2011.09.011 PMC3202986

[43] Renton A.E., Majounie E., Waite A., Simon-Sanchez J., Rollinson S., Gibbs J.R., Schymick J.C., Laaksovirta H., van Swieten J.C., Myllykangas L. et al. A hexanucleotide repeat expansion in C9ORF72 is the cause of chromosome 9p21-linked ALS-FTD. Neuron. 2011; 72:257–268. 21944779 10.1016/j.neuron.2011.09.010 PMC3200438

[44] Smeyers J., Banchi E.G., Latouche M. C9ORF72: what it is, what it does, and why it matters. Front. Cell Neurosci. 2021; 15:661447. 34025358 10.3389/fncel.2021.661447 PMC8131521

[45] van Blitterswijk M., DeJesus-Hernandez M., Niemantsverdriet E., Murray M.E., Heckman M.G., Diehl N.N., Brown P.H., Baker M.C., Finch N.A., Bauer P.O. et al. Association between repeat sizes and clinical and pathological characteristics in carriers of C9ORF72 repeat expansions (Xpansize-72): a cross-sectional cohort study. Lancet Neurol. 2013; 12:978–988. 24011653 10.1016/S1474-4422(13)70210-2 PMC3879782

[46] Nordin A., Akimoto C., Wuolikainen A., Alstermark H., Jonsson P., Birve A., Marklund S.L., Graffmo K.S., Forsberg K., Brannstrom T. et al. Extensive size variability of the GGGGCC expansion in C9orf72 in both neuronal and non-neuronal tissues in 18 patients with ALS or FTD. Hum. Mol. Genet. 2015; 24:3133–3142. 25712133 10.1093/hmg/ddv064

[47] van der Ende E.L., Jackson J.L., White A., Seelaar H., van Blitterswijk M., Van Swieten J.C. Unravelling the clinical spectrum and the role of repeat length in C9ORF72 repeat expansions. J. Neurol. Neurosurg. Psychiatry. 2021; 92:502–509. 33452054 10.1136/jnnp-2020-325377 PMC8053328

[48] Van Mossevelde S., van der Zee J., Cruts M., Van Broeckhoven C. Relationship between C9orf72 repeat size and clinical phenotype. Curr. Opin. Genet. Dev. 2017; 44:117–124. 28319737 10.1016/j.gde.2017.02.008

[49] O’Rourke J.G., Bogdanik L., Muhammad A., Gendron T.F., Kim K.J., Austin A., Cady J., Liu E.Y., Zarrow J., Grant S. et al. C9orf72 BAC transgenic mice display typical pathologic features of ALS/FTD. Neuron. 2015; 88:892–901. 26637796 10.1016/j.neuron.2015.10.027 PMC4672384

[50] Peters O.M., Cabrera G.T., Tran H., Gendron T.F., McKeon J.E., Metterville J., Weiss A., Wightman N., Salameh J., Kim J. et al. Human C9ORF72 hexanucleotide expansion reproduces RNA foci and dipeptide repeat proteins but not neurodegeneration in BAC transgenic mice. Neuron. 2015; 88:902–909. 26637797 10.1016/j.neuron.2015.11.018 PMC4828340

[51] Jiang J., Zhu Q., Gendron T.F., Saberi S., McAlonis-Downes M., Seelman A., Stauffer J.E., Jafar-Nejad P., Drenner K., Schulte D. et al. Gain of toxicity from ALS/FTD-linked repeat expansions in C9ORF72 is alleviated by antisense oligonucleotides targeting GGGGCC-containing RNAs. Neuron. 2016; 90:535–550. 27112497 10.1016/j.neuron.2016.04.006 PMC4860075

[52] Liu Y., Pattamatta A., Zu T., Reid T., Bardhi O., Borchelt D.R., Yachnis A.T., Ranum L.P. C9orf72 BAC mouse model with motor deficits and neurodegenerative features of ALS/FTD. Neuron. 2016; 90:521–534. 27112499 10.1016/j.neuron.2016.04.005

[53] Capecchi M.R. Altering the genome by homologous recombination. Science. 1989; 244:1288–1292. 2660260 10.1126/science.2660260

[54] Shimizu M., Gellibolian R., Oostra B.A., Wells R.D. Cloning, characterization and properties of plasmids containing CGG triplet repeats from the FMR-1 gene. J. Mol. Biol. 1996; 258:614–626. 8636996 10.1006/jmbi.1996.0273

[55] Ohshima K., Montermini L., Wells R.D., Pandolfo M. Inhibitory effects of expanded GAA.TTC triplet repeats from intron I of the Friedreich ataxia gene on transcription and replication in vivo. J. Biol. Chem. 1998; 273:14588–14595. 9603975 10.1074/jbc.273.23.14588

[56] Nair R.R., Tibbit C., Thompson D., McLeod R., Nakhuda A., Simon M.M., Baloh R.H., Fisher E.M.C., Isaacs A.M., Cunningham T.J. Sizing, stabilising, and cloning repeat-expansions for gene targeting constructs. Methods. 2021; 191:15–22. 32721467 10.1016/j.ymeth.2020.07.007 PMC8215685

[57] Valenzuela D.M., Murphy A.J., Frendewey D., Gale N.W., Economides A.N., Auerbach W., Poueymirou W.T., Adams N.C., Rojas J., Yasenchak J. et al. High-throughput engineering of the mouse genome coupled with high-resolution expression analysis. Nat. Biotechnol. 2003; 21:652–659. 12730667 10.1038/nbt822

[58] Osoegawa K., Tateno M., Woon P.Y., Frengen E., Mammoser A.G., Catanese J.J., Hayashizaki Y., de Jong P.J. Bacterial artificial chromosome libraries for mouse sequencing and functional analysis. Genome Res. 2000; 10:116–128. 10645956 PMC310499

[59] Osoegawa K., Mammoser A.G., Wu C., Frengen E., Zeng C., Catanese J.J., de Jong P.J. A bacterial artificial chromosome library for sequencing the complete human genome. Genome Res. 2001; 11:483–496. 11230172 10.1101/gr.169601 PMC311044

[60] Adams D.J., Quail M.A., Cox T., van der Weyden L., Gorick B.D., Su Q., Chan W.I., Davies R., Bonfield J.K., Law F. et al. A genome-wide, end-sequenced 129Sv BAC library resource for targeting vector construction. Genomics. 2005; 86:753–758. 16257172 10.1016/j.ygeno.2005.08.003

[61] Zhang Y., Buchholz F., Muyrers J.P., Stewart A.F. A new logic for DNA engineering using recombination in Escherichia coli. Nat. Genet. 1998; 20:123–128. 9771703 10.1038/2417

[62] Montasser M.E., Van Hout C.V., Miloscio L., Howard A.D., Rosenberg A., Callaway M., Shen B., Li N., Locke A.E., Verweij N. et al. Genetic and functional evidence links a missense variant in B4GALT1 to lower LDL and fibrinogen. Science. 2021; 374:1221–1227. 34855475 10.1126/science.abe0348

[63] Poueymirou W.T., Auerbach W., Frendewey D., Hickey J.F., Escaravage J.M., Esau L., Dore A.T., Stevens S., Adams N.C., Dominguez M.G. et al. F0 generation mice fully derived from gene-targeted embryonic stem cells allowing immediate phenotypic analyses. Nat. Biotechnol. 2007; 25:91–99. 17187059 10.1038/nbt1263

[64] Bram E., Javanmardi K., Nicholson K., Culp K., Thibert J.R., Kemppainen J., Le V., Schlageter A., Hadd A., Latham G.J. Comprehensive genotyping of the C9orf72 hexanucleotide repeat region in 2095 ALS samples from the NINDS collection using a two-mode, long-read PCR assay. Amyotroph. Lateral Scler. Frontotemporal Degener. 2019; 20:107–114. 30430876 10.1080/21678421.2018.1522353 PMC6513680

[65] Cleary E.M., Pal S., Azam T., Moore D.J., Swingler R., Gorrie G., Stephenson L., Colville S., Chandran S., Porteous M. et al. Improved PCR based methods for detecting C9orf72 hexanucleotide repeat expansions. Mol. Cell. Probes. 2016; 30:218–224. 27288208 10.1016/j.mcp.2016.06.001 PMC4978699

[66] Long A., Napierala J.S., Polak U., Hauser L., Koeppen A.H., Lynch D.R., Napierala M. Somatic instability of the expanded GAA repeats in Friedreich's ataxia. PLoS One. 2017; 12:e0189990. 29261783 10.1371/journal.pone.0189990 PMC5736210

[67] Giesselmann P., Brandl B., Raimondeau E., Bowen R., Rohrandt C., Tandon R., Kretzmer H., Assum G., Galonska C., Siebert R. et al. Analysis of short tandem repeat expansions and their methylation state with nanopore sequencing. Nat. Biotechnol. 2019; 37:1478–1481. 31740840 10.1038/s41587-019-0293-x

[68] Wheeler V.C., Auerbach W., White J.K., Srinidhi J., Auerbach A., Ryan A., Duyao M.P., Vrbanac V., Weaver M., Gusella J.F. et al. Length-dependent gametic CAG repeat instability in the Huntington's disease knock-in mouse. Hum. Mol. Genet. 1999; 8:115–122. 9887339 10.1093/hmg/8.1.115

[69] Duyao M., Ambrose C., Myers R., Novelletto A., Persichetti F., Frontali M., Folstein S., Ross C., Franz M., Abbott M. et al. Trinucleotide repeat length instability and age of onset in Huntington's disease. Nat. Genet. 1993; 4:387–392. 8401587 10.1038/ng0893-387

[70] Neto J.L., Lee J.M., Afridi A., Gillis T., Guide J.R., Dempsey S., Lager B., Alonso I., Wheeler V.C., Pinto R.M. Genetic contributors to intergenerational CAG repeat instability in Huntington's disease knock-in mice. Genetics. 2017; 205:503–516. 27913616 10.1534/genetics.116.195578 PMC5289832

[71] Jeggo P.A., Pearl L.H., Carr A.M. DNA repair, genome stability and cancer: a historical perspective. Nat. Rev. Cancer. 2016; 16:35–42. 26667849 10.1038/nrc.2015.4

[72] Tubbs A., Nussenzweig A. Endogenous DNA damage as a source of genomic instability in cancer. Cell. 2017; 168:644–656. 28187286 10.1016/j.cell.2017.01.002 PMC6591730

[73] Madabhushi R., Pan L., Tsai L.H. DNA damage and its links to neurodegeneration. Neuron. 2014; 83:266–282. 25033177 10.1016/j.neuron.2014.06.034 PMC5564444

[74] Baratz K.H., Tosakulwong N., Ryu E., Brown W.L., Branham K., Chen W., Tran K.D., Schmid-Kubista K.E., Heckenlively J.R., Swaroop A. et al. E2-2 protein and Fuchs's corneal dystrophy. N. Engl. J. Med. 2010; 363:1016–1024. 20825314 10.1056/NEJMoa1007064

[75] Fautsch M.P., Wieben E.D., Baratz K.H., Bhattacharyya N., Sadan A.N., Hafford-Tear N.J., Tuft S.J., Davidson A.E. TCF4-mediated Fuchs endothelial corneal dystrophy: insights into a common trinucleotide repeat-associated disease. Prog. Retin. Eye Res. 2021; 81:100883. 32735996 10.1016/j.preteyeres.2020.100883 PMC7988464

[76] Masnovo C., Lobo A.F., Mirkin S.M. Replication dependent and independent mechanisms of GAA repeat instability. DNA Repair (Amst.). 2022; 118:103385. 35952488 10.1016/j.dnarep.2022.103385 PMC9675320

[77] Foiry L., Dong L., Savouret C., Hubert L., te Riele H., Junien C., Gourdon G. Msh3 is a limiting factor in the formation of intergenerational CTG expansions in DM1 transgenic mice. Hum. Genet. 2006; 119:520–526. 16552576 10.1007/s00439-006-0164-7

[78] Liu L., Malkova A. Break-induced replication: unraveling each step. Trends Genet. 2022; 38:752–765. 35459559 10.1016/j.tig.2022.03.011 PMC9197877

[79] Mayle R., Campbell I.M., Beck C.R., Yu Y., Wilson M., Shaw C.A., Bjergbaek L., Lupski J.R., Ira G. DNA REPAIR. Mus81 and converging forks limit the mutagenicity of replication fork breakage. Science. 2015; 349:742–747. 26273056 10.1126/science.aaa8391 PMC4782627

[80] Nickoloff J.A., Sharma N., Taylor L., Allen S.J., Hromas R. The safe path at the fork: ensuring replication-associated DNA double-strand breaks are repaired by homologous recombination. Front. Genet. 2021; 12:748033. 34646312 10.3389/fgene.2021.748033 PMC8502867

[81] Deem A., Keszthelyi A., Blackgrove T., Vayl A., Coffey B., Mathur R., Chabes A., Malkova A. Break-induced replication is highly inaccurate. PLoS Biol. 2011; 9:e1000594. 21347245 10.1371/journal.pbio.1000594 PMC3039667

[82] Beck C.R., Carvalho C.M.B., Akdemir Z.C., Sedlazeck F.J., Song X., Meng Q., Hu J., Doddapaneni H., Chong Z., Chen E.S. et al. Megabase length hypermutation accompanies human structural variation at 17p11.2. Cell. 2019; 176:1310–1324. 30827684 10.1016/j.cell.2019.01.045 PMC6438178

[83] Costantino L., Sotiriou S.K., Rantala J.K., Magin S., Mladenov E., Helleday T., Haber J.E., Iliakis G., Kallioniemi O.P., Halazonetis T.D. Break-induced replication repair of damaged forks induces genomic duplications in human cells. Science. 2014; 343:88–91. 24310611 10.1126/science.1243211 PMC4047655

[84] Bhowmick R., Minocherhomji S., Hickson I.D. RAD52 facilitates mitotic DNA synthesis following replication stress. Mol. Cell. 2016; 64:1117–1126. 27984745 10.1016/j.molcel.2016.10.037

[85] Li S., Wang H., Jehi S., Li J., Liu S., Wang Z., Truong L., Chiba T., Wang Z., Wu X. PIF1 helicase promotes break-induced replication in mammalian cells. EMBO J. 2021; 40:e104509. 33470420 10.15252/embj.2020104509 PMC8047440

[86] Tsuzuki T., Fujii Y., Sakumi K., Tominaga Y., Nakao K., Sekiguchi M., Matsushiro A., Yoshimura Y., Morita T. Targeted disruption of the Rad51 gene leads to lethality in embryonic mice. Proc. Natl. Acad. Sci. U.S.A. 1996; 93:6236–6240. 8692798 10.1073/pnas.93.13.6236 PMC39005

[87] Pattamatta A., Nguyen L., Olafson H.R., Scotti M.M., Laboissonniere L.A., Richardson J., Berglund J.A., Zu T., Wang E.T., Ranum L.P.W. Repeat length increases disease penetrance and severity in C9orf72 ALS/FTD BAC transgenic mice. Hum. Mol. Genet. 2021; 29:3900–3918. 33378537 10.1093/hmg/ddaa279 PMC7906756

[88] Kalef-Ezra E., Edzeamey F.J., Valle A., Khonsari H., Kleine P., Oggianu C., Al-Mahdawi S., Pook M.A., Anjomani Virmouni S. A new FRDA mouse model [Fxn (null):YG8s(GAA) >800] with more than 800 GAA repeats. Front. Neurosci. 2023; 17:930422. 36777637 10.3389/fnins.2023.930422 PMC9909538

[89] Freudenreich C.H., Kantrow S.M., Zakian V.A. Expansion and length-dependent fragility of CTG repeats in yeast. Science. 1998; 279:853–856. 9452383 10.1126/science.279.5352.853

[90] Gadgil R.Y., Romer E.J., Goodman C.C., Rider S.D. Jr, Damewood F.J., Barthelemy J.R., Shin-Ya K., Hanenberg H., Leffak M Replication stress at microsatellites causes DNA double-strand breaks and break-induced replication. J. Biol. Chem. 2020; 295:15378–15397. 32873711 10.1074/jbc.RA120.013495 PMC7650239

[91] van den Broek W.J., Wansink D.G., Wieringa B. Somatic CTG*CAG repeat instability in a mouse model for myotonic dystrophy type 1 is associated with changes in cell nuclearity and DNA ploidy. BMC Mol. Biol. 2007; 8:61. 17645799 10.1186/1471-2199-8-61 PMC1940261

[92] Lee J.M., Pinto R.M., Gillis T., St Claire J.C., Wheeler V.C. Quantification of age-dependent somatic CAG repeat instability in Hdh CAG knock-in mice reveals different expansion dynamics in striatum and liver. PLoS One. 2011; 6:e23647. 21897851 10.1371/journal.pone.0023647 PMC3163641

[93] Dols-Icardo O., Garcia-Redondo A., Rojas-Garcia R., Sanchez-Valle R., Noguera A., Gomez-Tortosa E., Pastor P., Hernandez I., Esteban-Perez J., Suarez-Calvet M. et al. Characterization of the repeat expansion size in C9orf72 in amyotrophic lateral sclerosis and frontotemporal dementia. Hum. Mol. Genet. 2014; 23:749–754. 24057670 10.1093/hmg/ddt460

[94] Fratta P., Polke J.M., Newcombe J., Mizielinska S., Lashley T., Poulter M., Beck J., Preza E., Devoy A., Sidle K. et al. Screening a UK amyotrophic lateral sclerosis cohort provides evidence of multiple origins of the C9orf72 expansion. Neurobiol. Aging. 2015; 36:546.e1–546.e7. 10.1016/j.neurobiolaging.2014.07.037 PMC4270445 25179228

[95] Gijselinck I., Van Mossevelde S., van der Zee J., Sieben A., Engelborghs S., De Bleecker J., Ivanoiu A., Deryck O., Edbauer D., Zhang M. et al. The C9orf72 repeat size correlates with onset age of disease, DNA methylation and transcriptional downregulation of the promoter. Mol. Psychiatry. 2016; 21:1112–1124. 26481318 10.1038/mp.2015.159 PMC4960451

[96] Gijselinck I., Cruts M., Van Broeckhoven C. The genetics of C9orf72 expansions. Cold Spring Harb. Perspect. Med. 2018; 8:a026757. 28130313 10.1101/cshperspect.a026757 PMC5880162

[97] Jackson J.L., Finch N.A., Baker M.C., Kachergus J.M., DeJesus-Hernandez M., Pereira K., Christopher E., Prudencio M., Heckman M.G., Thompson E.A. et al. Elevated methylation levels, reduced expression levels, and frequent contractions in a clinical cohort of C9orf72 expansion carriers. Mol. Neurodegener. 2020; 15:7. 32000838 10.1186/s13024-020-0359-8 PMC6993399

[98] Shastri N., Tsai Y.C., Hile S., Jordan D., Powell B., Chen J., Maloney D., Dose M., Lo Y., Anastassiadis T. et al. Genome-wide identification of structure-forming repeats as principal sites of fork collapse upon ATR inhibition. Mol. Cell. 2018; 72:222–238. 30293786 10.1016/j.molcel.2018.08.047 PMC6407864

[99] Tubbs A., Sridharan S., van Wietmarschen N., Maman Y., Callen E., Stanlie A., Wu W., Wu X., Day A., Wong N. et al. Dual roles of poly(dA:dT) tracts in replication initiation and fork collapse. Cell. 2018; 174:1127–1142. 30078706 10.1016/j.cell.2018.07.011 PMC6591735

[100] Kaushal S., Freudenreich C.H. The role of fork stalling and DNA structures in causing chromosome fragility. Genes Chromosomes Cancer. 2019; 58:270–283. 30536896 10.1002/gcc.22721 PMC7083089

[101] Lokanga R.A., Kumari D., Usdin K. Common threads: aphidicolin-inducible and folate-sensitive fragile sites in the human genome. Front. Genet. 2021; 12:708860. 34567068 10.3389/fgene.2021.708860 PMC8456018

[102] Lopez-Gonzalez R., Lu Y., Gendron T.F., Karydas A., Tran H., Yang D., Petrucelli L., Miller B.L., Almeida S., Gao F.B. Poly(GR) in C9ORF72-related ALS/FTD compromises mitochondrial function and increases oxidative stress and DNA damage in iPSC-derived motor neurons. Neuron. 2016; 92:383–391. 27720481 10.1016/j.neuron.2016.09.015 PMC5111366

[103] Farg M.A., Konopka A., Soo K.Y., Ito D., Atkin J.D. The DNA damage response (DDR) is induced by the C9orf72 repeat expansion in amyotrophic lateral sclerosis. Hum. Mol. Genet. 2017; 26:2882–2896. 28481984 10.1093/hmg/ddx170

[104] Pham N., Yan Z., Yu Y., Faria Afreen M., Malkova A., Haber J.E., Ira G. Mechanisms restraining break-induced replication at two-ended DNA double-strand breaks. EMBO J. 2021; 40:e104847. 33844333 10.15252/embj.2020104847 PMC8126933

[105] Symington L.S. End resection at double-strand breaks: mechanism and regulation. Cold Spring Harb. Perspect. Biol. 2014; 6:a016436. 25085909 10.1101/cshperspect.a016436 PMC4107989

[106] Caldecott K.W. Causes and consequences of DNA single-strand breaks. Trends Biochem. Sci. 2024; 49:68–78. 38040599 10.1016/j.tibs.2023.11.001

[107] Whelan D.R., Lee W.T.C., Yin Y., Ofri D.M., Bermudez-Hernandez K., Keegan S., Fenyo D., Rothenberg E. Spatiotemporal dynamics of homologous recombination repair at single collapsed replication forks. Nat. Commun. 2018; 9:3882. 30250272 10.1038/s41467-018-06435-3 PMC6155164

[108] Gacy A.M., Goellner G., Juranic N., Macura S., McMurray C.T. Trinucleotide repeats that expand in human disease form hairpin structures in vitro. Cell. 1995; 81:533–540. 7758107 10.1016/0092-8674(95)90074-8

[109] Poggi L., Richard G.F. Alternative DNA structures in vivo: molecular evidence and remaining questions. Microbiol. Mol. Biol. Rev. 2021; 85:e00110-20. 33361270 10.1128/MMBR.00110-20 PMC8549851

[110] Lo Scrudato M., Poulard K., Sourd C., Tome S., Klein A.F., Corre G., Huguet A., Furling D., Gourdon G., Buj-Bello A. Genome editing of expanded CTG repeats within the human DMPK gene reduces nuclear RNA foci in the muscle of DM1 mice. Mol. Ther. 2019; 27:1372–1388. 31253581 10.1016/j.ymthe.2019.05.021 PMC6697452

[111] Meijboom K.E., Abdallah A., Fordham N.P., Nagase H., Rodriguez T., Kraus C., Gendron T.F., Krishnan G., Esanov R., Andrade N.S. et al. CRISPR/Cas9-mediated excision of ALS/FTD-causing hexanucleotide repeat expansion in C9ORF72 rescues major disease mechanisms in vivo and in vitro. Nat. Commun. 2022; 13:6286. 36271076 10.1038/s41467-022-33332-7 PMC9587249

[112] Kosicki M., Tomberg K., Bradley A. Repair of double-strand breaks induced by CRISPR-Cas9 leads to large deletions and complex rearrangements. Nat. Biotechnol. 2018; 36:765–771. 30010673 10.1038/nbt.4192 PMC6390938

[113] Owens D.D.G., Caulder A., Frontera V., Harman J.R., Allan A.J., Bucakci A., Greder L., Codner G.F., Hublitz P., McHugh P.J. et al. Microhomologies are prevalent at Cas9-induced larger deletions. Nucleic Acids Res. 2019; 47:7402–7417. 31127293 10.1093/nar/gkz459 PMC6698657

