# Supplementary material for: Somatic and intergenerational G4C2 hexanucleotide repeat instability in a human C9orf72 knock-in mouse model
Source: Nucleic Acids Res. 2024 Apr 10;52(10):5732–55. doi: 10.1093/nar/gkae250 (PMC11162798; doi:10.1093/nar/gkae250)
Supplement: gkae250_Supplemental_File [file gkae250_supplemental_file.pdf]

Supplementary figure 1

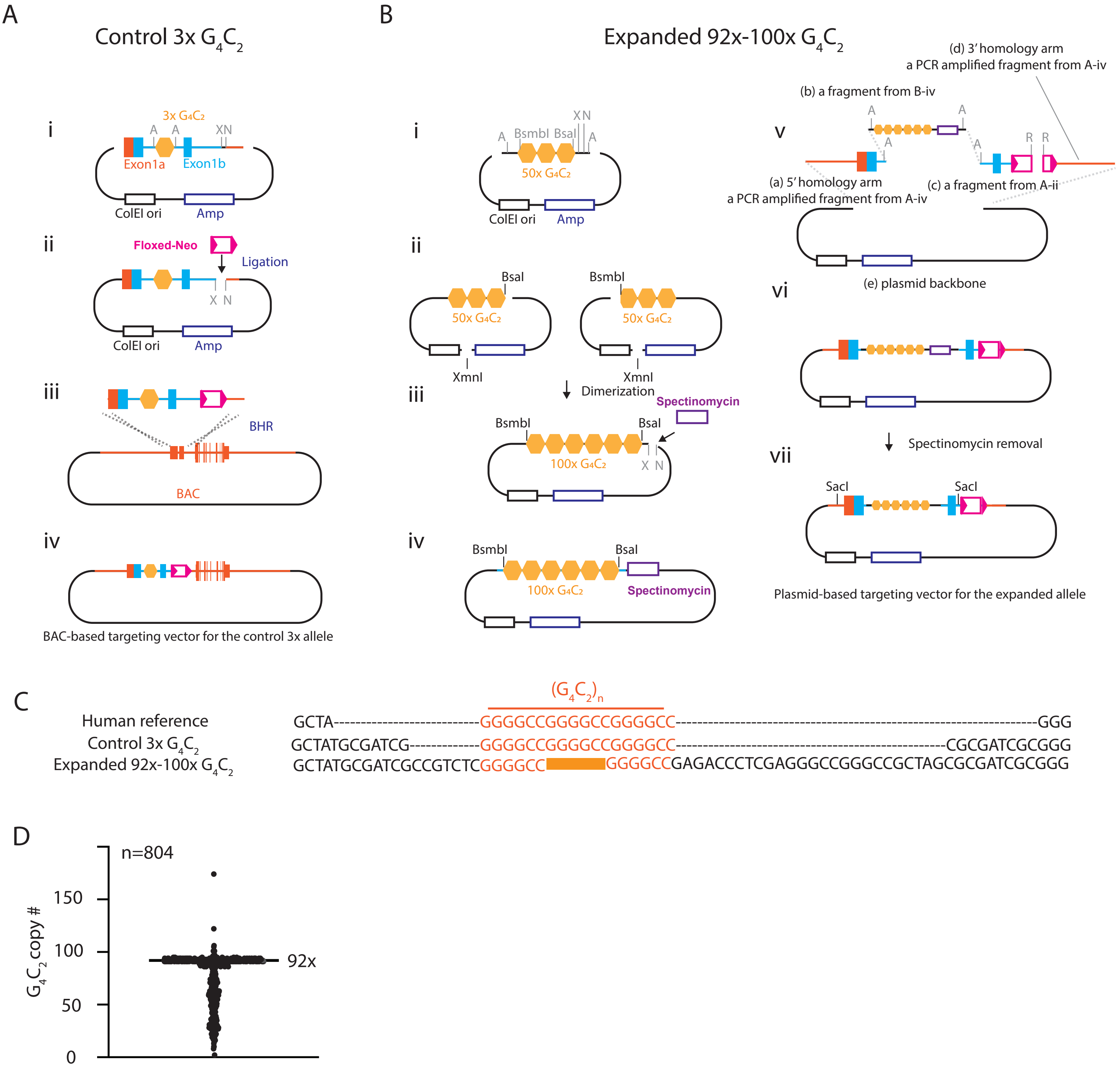

Supplementary Figure 1. Targeting vector construction for humanized *C9orf72* alleles.

See detailed description in **Materials and Methods** section. **A**. Construction procedures for control 3x G_4C_2 targeting vector. A, AsiSI. N, NheI. X, XhoI. **B**. Construction procedures for expanded 92-100x G_4C_2 targeting vector. **C**. Sequence comparison near the G_4C_2 repeats among human reference (hg38), control 3x G_4C_2 targeting vector and expanded 92x-100x G_4C_2 targeting vector. **D**. The G_4C_2 repeat length analysis in the expanded 92x-100x G_4C_2 targeting vector. n=804 nanopore sequencing reads were used for STRique analysis to determine the G_4C_2 repeat length. Most frequent repeat length call is shown as a horizontal bar and number.

Supplementary figure 2

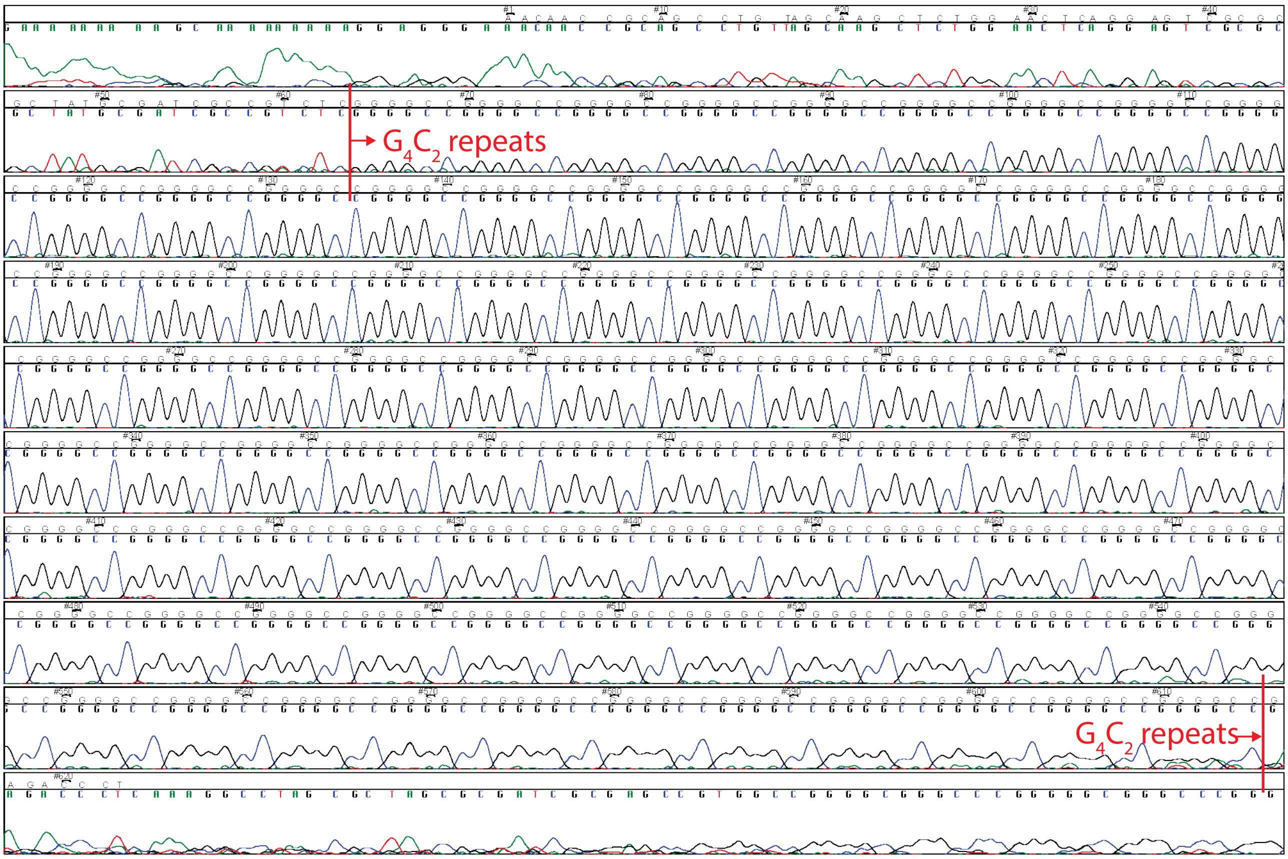

Supplementary Figure 2. Sanger sequencing chromatograph of the expanded G_4C_2 targeting vector.

Sanger sequencing results from the repeat expanded G_4C_2 targeting vector. 5' and 3' edges of the G_4C_2 repeats are indicated by red vertical lines.

Supplementary figure 3

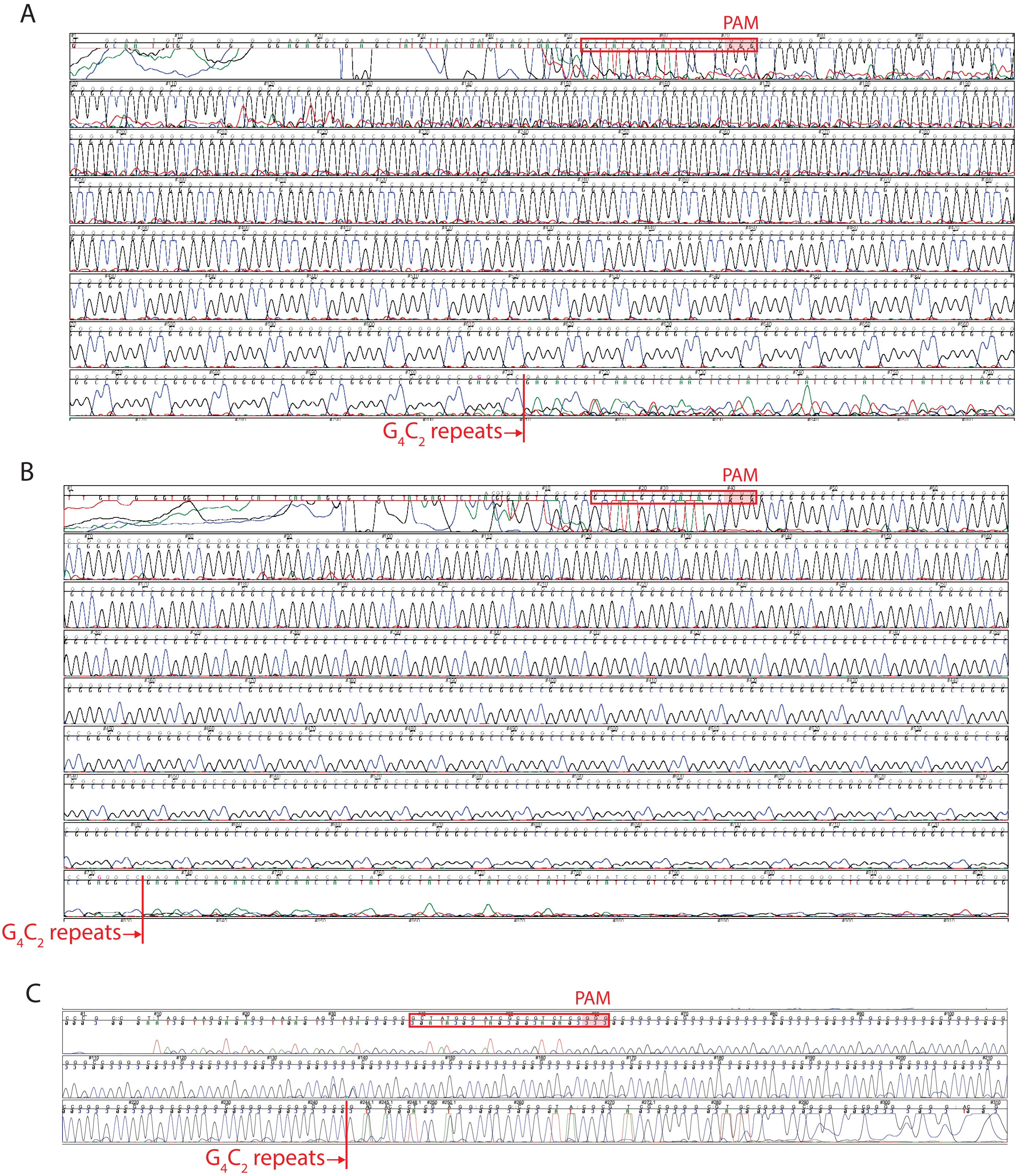

Supplementary Figure 3. Sanger sequencing chromatographs of the *C9orf72* G_4C_2 repeat-expanded clones. Representative repeat-expanded clones with 107 (**A**) and 117 (**B**) copies of G_4C_2 are shown. In both clones, small indels were generated by DSBs. Partial gRNA recognition and PAM sequences are shown in red squares. **C**, Sanger sequencing of *C9orf72*$^{hu31x/+}$ ES cell clone. gRNA recognition and PAM sequences are shown in red squares. The 3' edges of G_4C_2 repeats are indicated as red vertical lines.

Supplementary figure 4

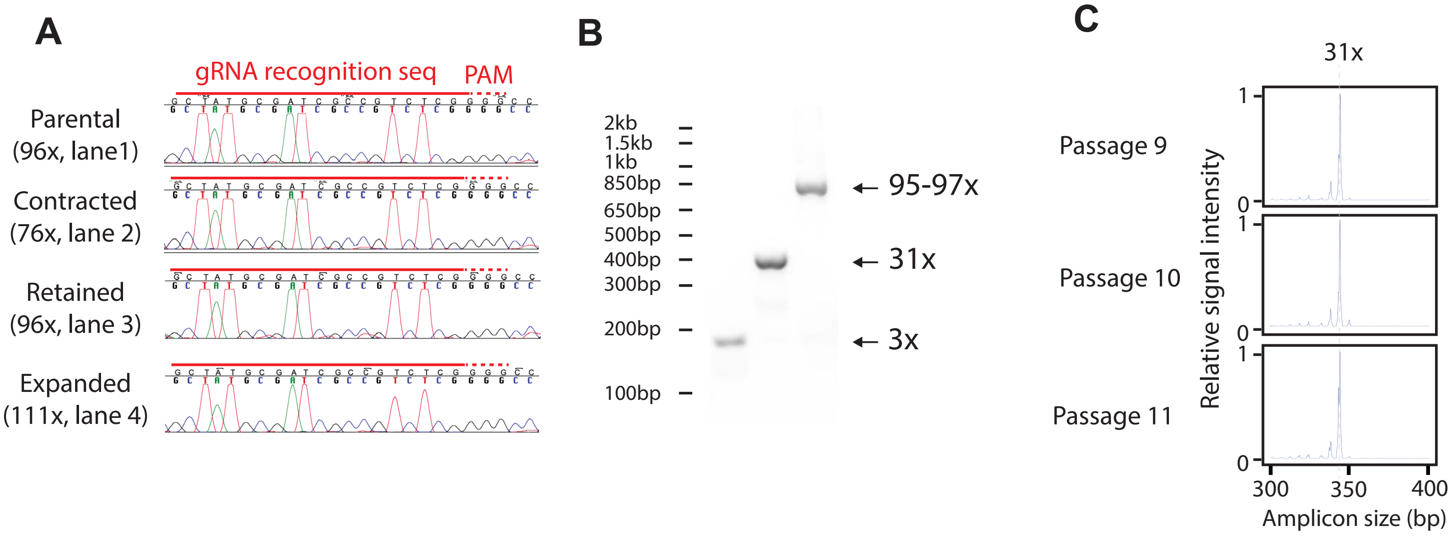

Supplementary Figure 4. **Sanger sequencing indel analysis of rearranged clones by Cas9-D10A nickase.**

A. Sanger sequence analysis of the Cas9-D10A nickase cleavage sites in representative clones. The G_4C_2 repeat length in each sample and corresponding lane number in **Figure 3H** are indicated in parentheses. **B**. two-primer PCR confirmation of G_4C_2 repeat length in targeted clones. **C**, RP-PCR analysis of the *C9orf72*$^{hu31x/+}$ ES cells over passages (passages 9-11). Capillary electrophoresis (CE) traces of gene-specific PCR products shown.

Supplementary figure 5

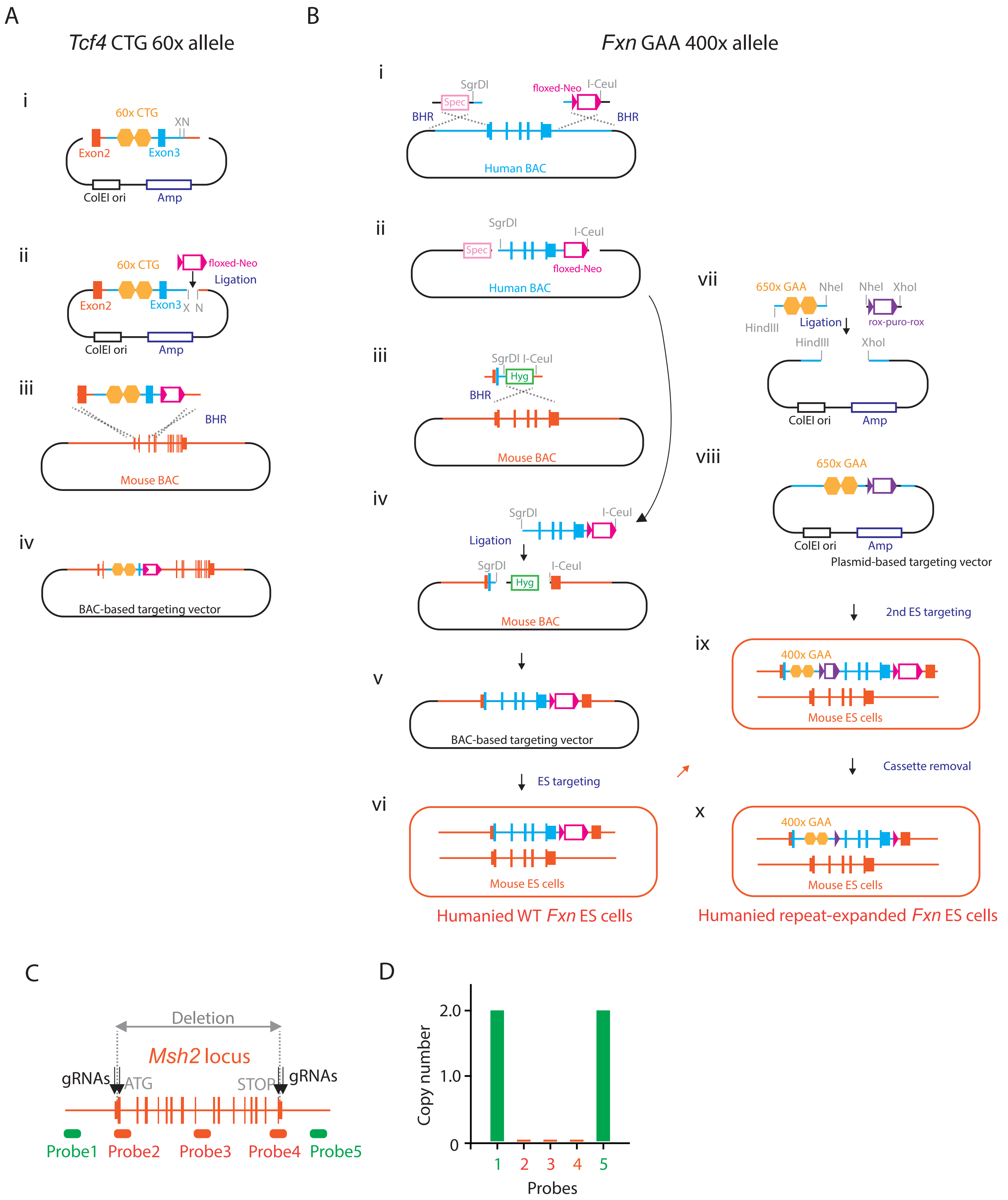

Supplementary Figure 5. Targeting strategies for humanized *Tcf4* and humanized *Fxn* alleles.

A. Construction procedures for the humanized *Tcf4* with 60x CTG targeting vector. See Materials and Methods section for detail. **B**. Generation of the humanized *Fxn* with 400x GAA repeats. Two rounds of gene targeting were performed to generate the allele. First, a BAC-based targeting vector that contained WT human *FXN*, from ATG to 3' UTR, and mouse homology arms for homologous recombination was generated (i-v). The human sequence in this vector was inserted in mouse genome by gene targeting (v-vi). Next, a plasmid-based targeting vector that contained 650x GAA repeats was generated (vii-viii). This expanded GAA repeats were inserted in the humanized *Fxn* locus by second gene targeting to obtain the Friedreich's ataxia model (viii-x). The largest repeat length among targeted clones was 400x. We used this 400x GAA clone is this study. **C.** *Msh2* Crispr KO strategy using four gRNA and WT Cas9. Three internal probes were designed to confirm the deletion of the locus, at 5' end, middle, and 3' end of the deleted region (probe 2, 3 and 4 respectively). Two external probes (probes 1 and 5) were designed to confirm that outside of the target locus was intact. **D**. Copy number counting using the five Taqman probes. Two copies outside of *Msh2* locus (probes 1 and 5) and zero copy inside of the locus (probes 2-4) confirmed the deletion of the locus.

Supplementary figure 6

A

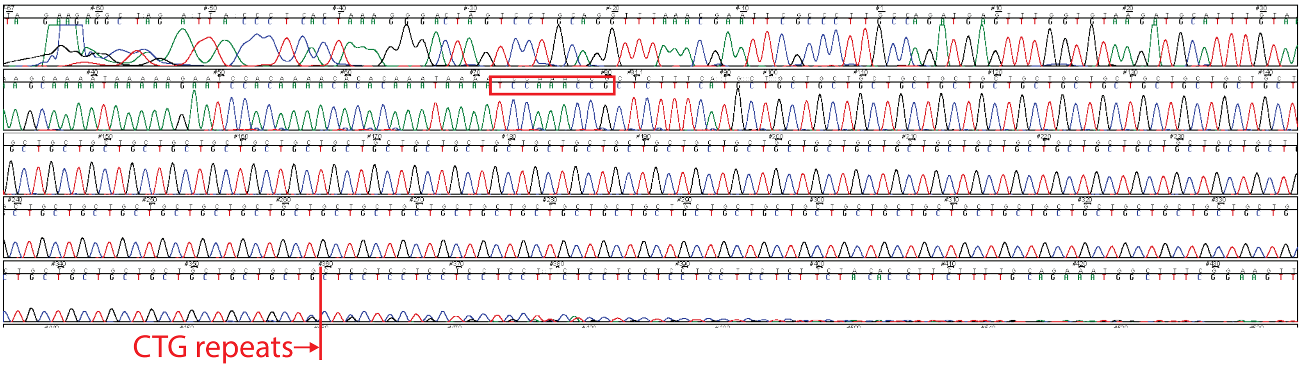

B

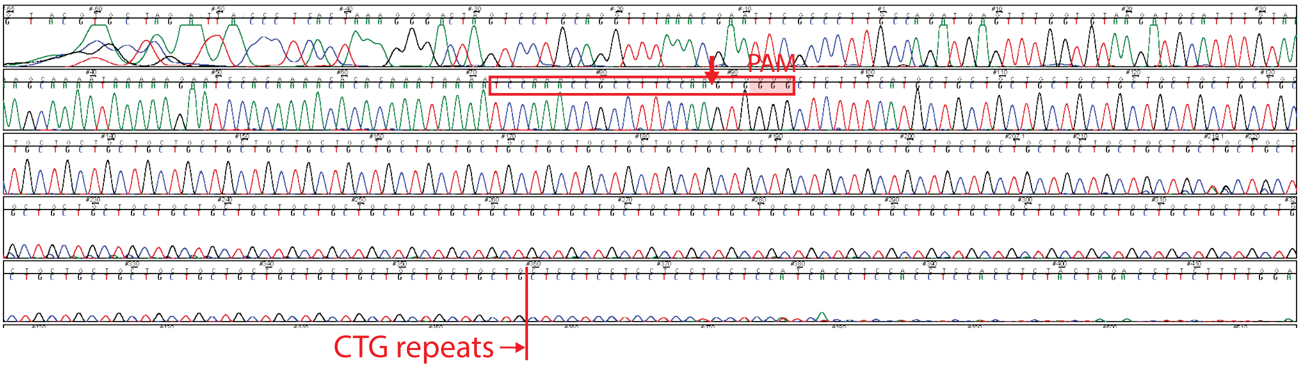

Supplementary Figure 6. Sanger sequencing chromatographs of the *Tcf4* CTG repeat-expanded clones. The two largest repeat-expanded clones with 86x (**A**) or 87x (**B**) CTG repeats. 3' edges of the CTG repeats are indicated as red vertical lines. The clone in **A** (corresponding to **Figure 4B** lane 2 top band) received small deletion after a DSB. A partial gRNA recognition sequence indicated as a red square. The clone in **B** (corresponding to **Figure 4B** lane 3) retained the intact gRNA protospacer and PAM sequences indicated as a red square.

Supplementary figure 7

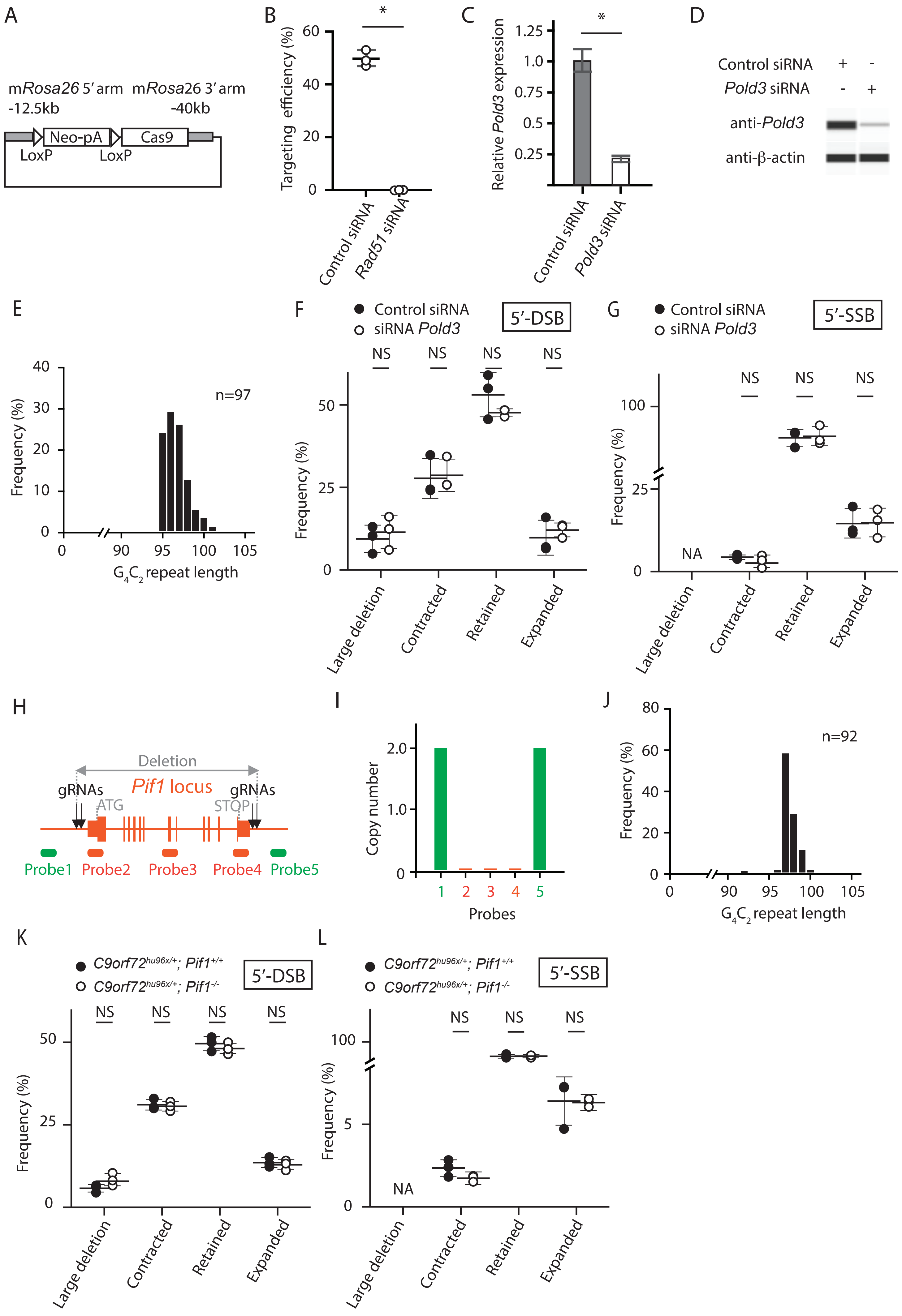

Supplementary Figure 7. Mechanistic analysis of DSB/SSB-induced repeat expansions.

A. Structure of the BAC-based *Rosa26* KI targeting vector. **B**. Targeting efficiency of the BAC-based *Rosa26* KI with siRNA *Rad51*. Three independent experiments were performed. * P<0.05. *Pold3* siRNA efficiency assessed by mRNA (**C**) and protein (**D**) levels**. E**. Repeat length distribution of *Pold3* siRNA-treated *C9orf72*$^{hu96x/+}$ cells after subcloning in the non-perturbed condition. Frequencies of the indicated changes after 5'-DSB (**F**) or 5'-SSB (**G**) introduction with *Pold3* siRNA in *C9orf72*$^{hu96x/+}$ ES cells. **H**. *Pif1* Crispr KO strategy using four gRNA and WT Cas9. Three internal probes were designed to confirm the deletion of the locus, at 5' end, middle, and 3' end of the deleted region (probe 2, 3 and 4 respectively). Two external probes (probes 1 and 5) were designed to confirm that outside of the target locus was intact. **I**. Copy number counting using the five Taqman probes. Two copies outside of *Pif1* locus (probes 1 and 5) and zero copy inside of the locus (probes 2-4) confirmed the deletion of the locus. **J**. Repeat length distribution of *C9orf72*$^{hu96x/+}$;*Pif1*$^{-/-}$ ES cells after subcloning in non-perturbed condition. Frequencies of the indicated changes after 5'-DSB (**K**) or 5'-SSB (**L**) introduction in *C9orf72*$^{hu96x/+}$;*Pif1*$^{-/-}$ ES cells. NS, not significant. NA, not applicable.

Supplementary figure 8

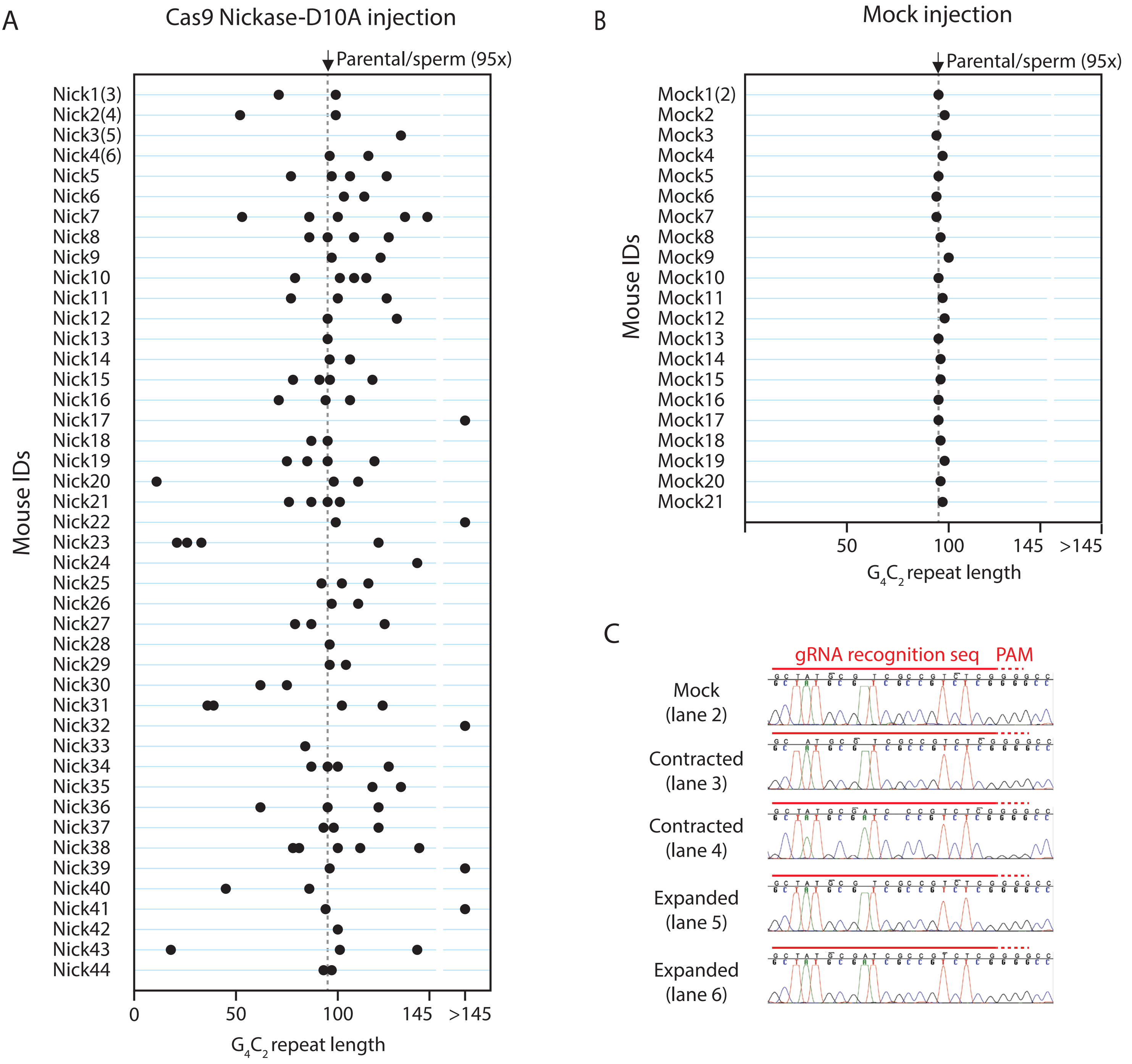

Supplementary Figure 8. **Characterization of the genome rearrangement in tails induced by Cas9-D10A nickase one-cell embryo injection.**

The G_4C_2 repeat species detected in tails from the mice that received Cas9-D10A nickase and C9-5' gRNA (**A**) or mock gRNA (**B**) at one-cell embryo stage. Each horizontal blue line represents one mouse. 44 and 21 mice were analyzed from nickase and mock injection respectively. Black-filled circles indicate G_4C_2 repeat species; most of mice that received nickase exhibited mosaicism. Mock1 and Nick1-4 samples correspond to the samples in lanes 2 and 3-6 (lane numbers shown in parentheses) in **Figure 7B** respectively. The dotted lines indicate the repeat length in the parental sperm sample (95 copies of G_4C_2). **C**. Sanger sequence analysis of the Cas9-D10A nickase cleavage sites. Corresponding lane numbers in **Figure 7B** are indicated in parentheses.

Supplementary figure 9

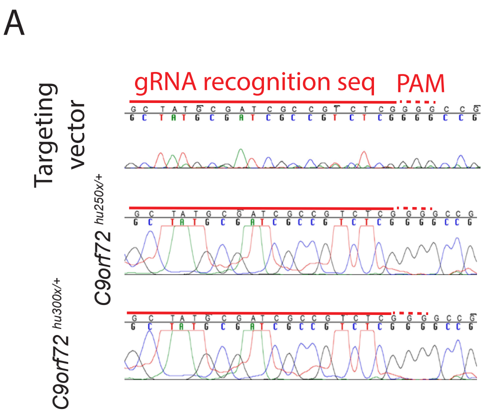

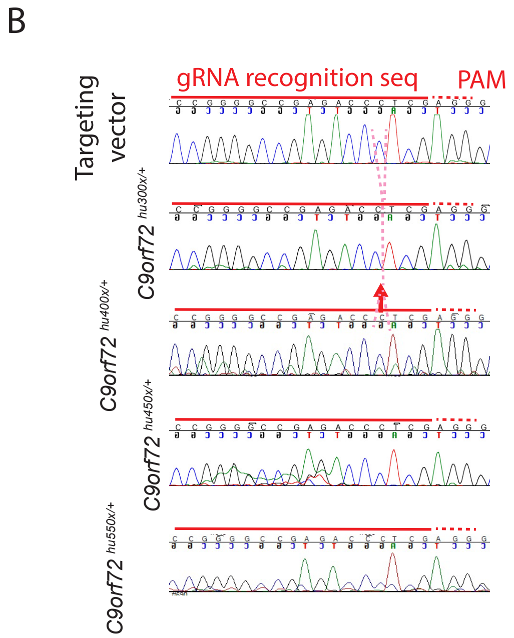

Supplementary Figure 9. Characterization of the large *C9orf72* G_4C_2 repeat alleles.

A-**B.** Indel analysis of 5'- (**A**) or 3'- (**B**) DSB site in the indicated mES cell clones. gRNA recognition sequence and PAM sequence are indicated by solid and dotted red lines respectively. A single bp deletion at 3'-DSB in *C9orf72*$^{hu300x/+}$ clone is indicated with a red arrow.

Supplementary Figure 10

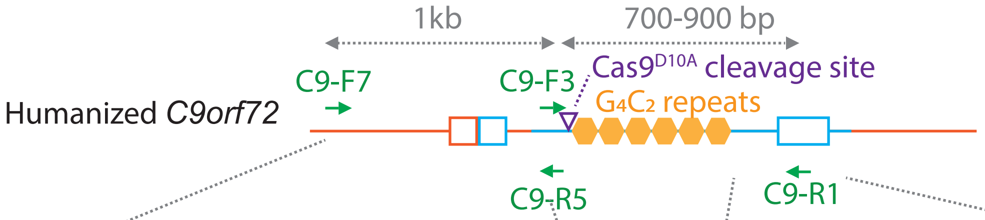

5′ upstream

```
Parental clone     1 AGATATACATCTGAAGACCACGGTACAAAACACTGTAAGAACTTGATAGAATGACAACTG   60
Repeat expanded    1 AGATATACATCTGAAGACCACGGTACAAAACACTGTAAGAACTTGATAGAATGACAACTG   60
                     ************************************************************

Parental clone    61 CTAGGTAAAAAAAAAAAAAAAAAAAAAAAAAAAAAAAAAAAAAAGCATACAATACCTGGT  120
Repeat expanded   61 CTAGGTAAAAAAAAAAAAAAAAAAAAAAAAAAAAAAAAAAAAAAGCATACAATACCTGGT  120
                     ************************************************************

Parental clone   121 GAGAGTTCTATTTTTACCGAAGGTGGTATTGATAGGTATTCTGTTATTAATGCCTTTCTT  180
Repeat expanded  121 GAGAGTTCTATTTTTACCGAAGGTGGTATTGATAGGTATTCTGTTATTAATGCCTTTCTT  180
                     ************************************************************

Parental clone   181 TTCCCTATAAATGATGAAAAGTTGCTGGAAAATAATAAACACTACTCATCTGTAGTGAAA  240
Repeat expanded  181 TTCCCTATAAATGATGAAAAGTTGCTGGAAAATAATAAACACTACTCATCTGTAGTGAAA  240
                     ************************************************************

Parental clone   241 AGCCACAATACAGTTACAAACCAATCAATCAATCAATAAATCAGACGTCATGGTGTTCTT  300
Repeat expanded  241 AGCCACAATACAGTTACAAACCAATCAATCAATCAATAAATCAGACGTCATGGTGTTCTT  300
                     ************************************************************

Parental clone   301 TTCCCAAAGGTTAAAAAACAAAGTGCACTGTGCTATTTGGCAAAAATGACGTTTAGAAGA  360
Repeat expanded  301 TTCCCAAAGGTTAAAAAACAAAGTGCACTGTGCTATTTGGCAAAAATGACGTTTAGAAGA  360
                     ************************************************************

Parental clone   361 AAACACGGTGACTACGCACAGAGGGTGGGGGAATCATTGTGCTTGTTGCGGAGTGAACAC  420
Repeat expanded  361 AAACACGGTGACTACGCACAGAGGGTGGGGGAATCATTGTGCTTGTTGCGGAGTGAACAC  420
                     ************************************************************

Parental clone   421 GTACAGTGTGCACGCAGACTTACGGCATTTAACCGTGTCATAGGGACCAAAGGAAATCCA  480
Repeat expanded  421 GTACAGTGTGCACGCAGACTTACGGCATTTAACCGTGTCATAGGGACCAAAGGAAATCCA  480
                     ************************************************************

Parental clone   481 CTCACTCACTAAATATTTGTTGAGCACCCACTACCTGCCAACTCCCAAACAAAACAAAGC  540
Repeat expanded  481 CTCACTCACTAAATATTTGTTGAGCACCCACTACCTGCCAACTCCCAAACAAAACAAAGC  540
                     ************************************************************

Parental clone   541 AAAACTACTTACAACCACAAACTACGCTTCGTAACCTAGATAGATAACGCAGGTGACACT  600
Repeat expanded  541 AAAACTACTTACAACCACAAACTACGCTTCGTAACCTAGATAGATAACGCAGGTGACACT  600
                     ************************************************************

Parental clone   601 ATCTATCTAGGTTGAGCTCAGCTCTGCCCATGCTTTTCCTGAGCGGCTCTTGGAAGAAAA  660
Repeat expanded  601 ATCTATCTAGGTTGAGCTCAGCTCTGCCCATGCTTTTCCTGAGCGGCTCTTGGAAGAAAA  660
                     ************************************************************

Parental clone   661 GCTACAAAGCCCATGACAGCCTCCGCCTGGCCAGCTGCCACTGGCATCTCAAGGCTGGCA  720
Repeat expanded  661 GCTACAAAGCCCATGACAGCCTCCGCCTGGCCAGCTGCCACTGGCATCTCAAGGCTGGCA  720
                     ************************************************************

Parental clone   721 AAGCAAAGTGAAAGCGCCAACCCGGAACTTACGGAGTCCCACGAGGGAACCGCGGCGCGT  780
Repeat expanded  721 AAGCAAAGTGAAAGCGCCAACCCGGAACTTACGGAGTCCCACGAGGGAACCGCGGCGCGT  780
                     ************************************************************

Parental clone   781 CAAGCAGAGACGAGTTCCGCCCACGTGAAAGATGGCGTTTGTAGTGACAGCCATCCCAAT  840
Repeat expanded  781 CAAGCAGAGACGAGTTCCGCCCACGTGAAAGATGGCGTTTGTAGTGACAGCCATCCCAAT  840
                     ************************************************************

Parental clone   841 TGCCCTTTCCTTCTAGGTGGAAAGTGGGGTCTAGCAAGAGCAGGTGTGGGTTTAGGAGGT  900
Repeat expanded  841 TGCCCTTTCCTTCTAGGTGGAAAGTGGGGTCTAGCAAGAGCAGGTGTGGGTTTAGGAGGT  900
                     ************************************************************

Parental clone   901 GTGTGTTTTTGTTTTTCCCACCCTCTCTCCCCACTACTTGCTCTCACAGTACTCGCTGAG  960
Repeat expanded  901 GTGTGTTTTTGTTTTTCCCACCCTCTCTCCCCACTACTTGCTCTCACAGTACTCGCTGAG  960
                     ************************************************************

Parental clone   961 GGTGAACAAGAAAAGACCTGATAAAGATTAACCAGAAGAAAACAAGGAGGGAAACAA 1017
Repeat expanded  961 GGTGAACAAGAAAAGACCTGATAAAGATTAACCAGAAGAAAACAAGGAGGGAAACAA 1017
                     *********************************************************
```

3′ upstream

```
Parental clone     1 GAGACCCTCGAGGGCCGGCCGCTAGCGCGATCGCGGGGCGTGGTCGGGGCGGGCCCGGGG  60
Repeat expanded    1 GAGACCCTCGAGGGCCGGCCGCTAGCGCGATCGCGGGGCGTGGTCGGGGCGGGCCCGGGG  60
                     ************************************************************

Parental clone    61 GCGGGCCCGGGGCGGGGCTGCGGTTGCGGTGCCTGCGCCCGCGGCGGCGGAGGCGCAGGC 120
Repeat expanded   61 GCGGGCCCGGGGCGGGGCTGCGGTTGCGGTGCCTGCGCCCGCGGCGGCGGAGGCGCAGGC 120
                     ************************************************************

Parental clone   121 GGTGGCGAGTGGGTGAGTGAGG 142
Repeat expanded  121 GGTGGCGAGTGGGTGAGTGAGG 142
                     **********************
```

Supplementary Figure 10. Sequencing SSB-induced repeat-expanded clone.

5' upstream of Cas9-D10A cleavage site (approximately 1kb), and 3' downstream including G_4C_2 repeats (approximately 700-900bp) were PCR amplified from SSB-induced repeat expanded clones using primer pair C9-F7/C9-R5 or C9-F3/C9-R1, and the amplicons were nanopore sequenced. Consensus sequences of the expanded clones were aligned with the parental sequence. Example from one of the clones shown.

ROI	Name	Strand	Protospacer	PAM
C9orf72	C9-5’	+	GCTATGCGATCGCCGTCTCG	GGG
C9orf72	C9-3’	+	GGCCGGGGCCGAGACCCTCG	AGG
C9orf72	C9-ONT-1	+	AGCCCATGACAGCCTCCGCC	TGG
C9orf72	C9-ONT-2	+	ACACGGTGACTACGCACAGA	GGG
C9orf72	C9-ONT-3	-	CTGGCCAGACAGACCCTGTG	TGG
C9orf72	C9-ONT-4	-	CAAGACCACTGTAAAGACAG	TGG
Tcf4	Tcf4-5’	+	TCCAAACCGCCTTCCAAGTG	GGG
Fxn	Fxn-3’	+	GAAAATAAAGAAAAGTTAGC	CGG
Msh2	Msh2-1	+	GTGGAGCGGCTCGGAAATGG	CGG
Msh2	Msh2-2	-	TCCAACTGCAGCGTCTCCTT	AGG
Msh2	Msh2-3	+	TCGTAAACGAGATCATTTCA	CGG
Msh2	Msh2-4	-	GCCCACCCACTGTCCCCTCA	CGG
Pif1	Pif1-1	-	GTGACAGTTACTCTCCTGGT	TGG
Pif1	Pif1-2	+	GAGAGGCCGTGTTTAGGATA	CGG
Pif1	Pif1-3	-	TTCCACGAGTGGGCGTGGTG	GGG
Pif1	Pif1-4	+	AGACCAGGCCGGTGATAGGG	TGG

Supplementary table 1. sgRNA used in this study.

ROI	Name	Sequence
C9orf72	C9-F1	CGCAGCCTGTAGCAAGCTCTGGAACTCAGGAGTCG
C9orf72	C9-F2	AGCAAGCTCTGGAACTCAGGAGTCG
C9orf72	C9-F3	Sequence not disclosed
C9orf72	C9-F4	Sequence not disclosed
C9orf72	C9-F5	CTGTAGCAAGCTCTGGAACTCAGGAGTCG
C9orf72	C9-F6	TACGCATCCCAGTTTGAGACGGGCCGGGGCCGGGGCCGG
C9orf72	C9-F7	TGTGGATTAGCTGACTGGCC
C9orf72	C9-R1	TGCGCCTCCGCCGCCGCGGGCGCAGGCACCGCAACCGCA
C9orf72	C9-R2	CCTCACTCACCCACTCGCCAC
C9orf72	C9-R3	TACGCATCCCAGTTTGAGACGCCCCGGCCCCGGCCCCGGCCCC
C9orf72	C9-R4	CGGGCGCAGGCACCGCAACC
C9orf72	C9-R5	TACAGGCTGCGGTTGTTTCC
C9orf72	C9-tail	TACGCA TCCCAG TTTGAGACG
Tcf4	Tcf4-F1	GCCAGATGAGTTTGGTGTAAG
Tcf4	Tcf4-R1	CACGTTGTTCGTCTTTCC
Fxn	Fxn-F1	GGCTTGAACTTCCCACACGTGTT
Fxn	Fxn-R1	AGGACCATCATGGCCACACTT

Supplementary table 2. Primers used in this study.

ROI	Probe	Oligo	Sequence
Msh2	Probe 1	Forward	TGGTGTGAATCGGGATAGACTATTC
Msh2	Probe 1	Probe	AGGCTCATTCACTGTTCAACCGCCA
Msh2	Probe 1	Reverse	TCCTCATGAAGTTGTAGAATCACAA
Msh2	Probe 2	Forward	AGGGTCGCTCTGTGCTCTAGTG
Msh2	Probe 2	Probe	CTCGGAAATGGCGGTGCAGCCT
Msh2	Probe 2	Reverse	TCCAACTGCAGCGTCTCCTT
Msh2	Probe 3	Forward	AGGGCTGTGCTGAGGATGAG
Msh2	Probe 3	Probe	AAAGCCACAGAGCAGCATGCCC
Msh2	Probe 3	Reverse	CAGGAACCAACAATGTGCAAGAAG
Msh2	Probe 4	Forward	CCGAGGTGGTCGCAAAGAAC
Msh2	Probe 4	Probe	CAGCTTCGTAAACGAGATCATTTCACGG
Msh2	Probe 4	Reverse	ACGGAGCCGGAGCCTTTA
Msh2	Probe 5	Forward	GCTACGGTGGGTTGAGTTGTTAG
Msh2	Probe 5	Probe	CACCGAGTCGGAGACAACACGC
Msh2	Probe 5	Reverse	CCCAGCTTCCTTTAGAGTATGGAA
Pif1	Probe 1	Forward	GCGGCAGCAAGCAGAGAATC
Pif1	Probe 1	Probe	ACACGTGCACCCAACCCAGAGC
Pif1	Probe 1	Reverse	GGCGGAGCCAGAGTGAGT
Pif1	Probe 2	Forward	TCGCTCCAGAGTGCTTGGAAT
Pif1	Probe 2	Probe	TGGCACCTTCTGTTACCAGTGACA
Pif1	Probe 2	Reverse	GCGGTGACGGGAAATCCT
Pif1	Probe 3	Forward	AGCCACGAGACTATGTACCCAT
Pif1	Probe 3	Probe	CAGGATGATGTGGCCCTGACCA
Pif1	Probe 3	Reverse	GGCCCAAGCCTTCTATTTACC
Pif1	Probe 4	Forward	CAGACACAAGCCCAAGATAGAAGT
Pif1	Probe 4	Probe	TGCTCAGCCTAGCACAGCCC
Pif1	Probe 4	Reverse	CAGTCGGGCTCTGAATGATGT
Pif1	Probe 5	Forward	GCCGAACTGAGTCCTTTACAAAC
Pif1	Probe 5	Probe	TGCCGACTTGGCTCGACAAAGT
Pif1	Probe 5	Reverse	GCCGTCCCACAAACATGGAG

Supplementary table 3. Taqman probes used in this study.
